# Insights into *KIF11* pathogenesis in microcephaly-lymphedema-chorioretinopathy syndrome from a lymphatic perspective

**DOI:** 10.1172/jci.insight.177656

**Published:** 2025-12-18

**Authors:** Kazim Ogmen, Sara E. Dobbins, Rose Yinghan Behncke, Ines Martinez-Corral, Ryan C.S. Brown, Michelle Meier, Sascha Ulferts, Nils Rouven Hansmeier, Ege Sackey, Ahlam Alqahtani, Christina Karapouliou, Dionysios Grigoriadis, Juan C. Del Rey Jimenez, Michael Oberlin, Denise Williams, Arzu Ekici, Kadri Karaer, Steve Jeffery, Peter Mortimer, Kristiana Gordon, Kazuhide S. Okuda, Benjamin M. Hogan, Taija Mäkinen, René Hägerling, Sahar Mansour, Silvia Martin-Almedina, Pia Ostergaard

**Affiliations:** 1 School of Health & Medical Sciences, City St George’s, University of London, London, United Kingdom.; 2 Research Group Lymphovascular Medicine and Translational 3D-Histopathology, Institute of Medical and Human Genetics, Charité - Universitätsmedizin Berlin, Germany.; 3 Berlin Institute of Health at Charité - Universitätsmedizin Berlin, BIH Center for Regenerative Therapies, Berlin, Germany.; 4 Department of Immunology, Genetics and Pathology, Uppsala University, Sweden.; 5 Organogenesis and Cancer Program, Peter MacCallum Cancer Centre, Melbourne, Victoria, Australia.; 6 Sir Peter MacCallum Department of Oncology and Department of Anatomy and Physiology, University of Melbourne, Victoria, Australia.; 7 Bioinformatics Core Facility, Peter MacCallum Cancer Centre, Melbourne, Victoria, Australia.; 8 Research Group Development and Disease, Max Planck Institute for Molecular Genetics, Berlin, Germany.; 9 South West Thames Centre for Genomics, St George’s University Hospitals NHS Foundation Trust, London, United Kingdom.; 10 Bioscience Institute, Wolfson Childhood Cancer Research Centre, Brewery Lane, Newcastle University, Newcastle upon Tyne, United Kingdom.; 11 Földiklinik GmbH & Co. KG, European Center for Lymphology Black Forest, Hinterzarten, Germany.; 12 The National Congenital Anomaly and Rare Diseases Registration Services (NCARDRS) NHS England, Birmingham Government Hub, Birmingham, United Kingdom.; 13 University of Health Sciences, Bursa Yüksek Īhtisas Training and Research Hospital, Department of Pediatric Neurology, Bursa, Turkey.; 14 Department of Medical Genetics, Pamukkale University Faculty of Medicine, Denizli, Turkey.; 15 Dermatology & Lymphovascular Medicine, St George’s University Hospitals NHS Foundation Trust, London, United Kingdom.; 16 Translational Cancer Medicine Program and Department of Biochemistry and Developmental Biology, University of Helsinki, Helsinki, Finland.; 17 Wihuri Research Institute, Helsinki, Finland.

**Keywords:** Clinical Research, Genetics, Vascular biology, Cardiovascular disease, Molecular genetics

## Abstract

Pathogenic variants in kinesin *KIF11* underlie microcephaly-lymphedema-chorioretinopathy (MLC) syndrome. Although well known for regulating spindle dynamics ensuring successful cell division, the association of *KIF11* (encoding EG5) with development of the lymphatic system and how *KIF11* pathogenic variants lead to lymphatic dysfunction and lymphedema remain unknown. Using patient-derived lymphoblastoid cells, we demonstrated that patients with MLC carrying pathogenic stop-gain variants in *KIF11* have reduced mRNA and protein levels. Lymphoscintigraphy showed reduced tracer absorption, and intestinal lymphangiectasia was detected in one patient, pointing to impairment of lymphatic function caused by *KIF11* haploinsufficiency. We revealed that *KIF11* is expressed in early human and mouse development with the lymphatic markers VEGFR3, podoplanin, and PROX1. In zebrafish, single-cell RNA-Seq identified *kif11* specifically expressed in endothelial precursors. In human lymphatic endothelial cells, EG5 inhibition with ispinesib reduced VEGFC-driven AKT phosphorylation, migration, and spheroid sprouting. *KIF11* knockdown reduced *PROX1* and *VEGFR3* expression, providing for the first time to our knowledge a link between *KIF11* and drivers of lymphangiogenesis and lymphatic identity.

## Abstract 2

Ogmen et al., studies how pathogenic variants in kinesin *KIF11* underlie microcephaly-lymphedema-chorioretinopathy (MLC) syndrome, how *KIF11* (encoding EG5) associates with the development of the lymphatic system, and how *KIF11* pathogenic variants lead to lymphatic dysfunction and lymphedema.

## Introduction

Microcephaly-lymphedema-chorioretinopathy syndrome (MLC; ORPHA:2526), also known as microcephaly with or without chorioretinopathy, lymphedema, or intellectual disability syndrome, is an autosomal dominant condition in which about 50% of patients present with congenital primary lymphedema (1). Variants in *KIF11* cause 75% of MLC cases (1–3). The pathogenic basis of *KIF11*-associated retinal vascular disease has been investigated through the generation of a mouse model for familial exudative vitreoretinopathy (4). More recently, the phenotypic spectrum of *KIF11*-associated MLC has been expanded to include renal system involvement (5). However, how *KIF11* variants lead to lymphedema, the specific hallmark of lymphatic dysfunction, is not known.

*KIF11* encodes EG5, a motor protein essential in cell-cycle dynamics. EG5 inhibition arrests cells in metaphase, inducing monopolar spindles in vitro (6, 7). No phenotype was found in heterozygous mice (*Eg5*^+/–^), but homozygous deletion (*Eg5*^−/−^) was lethal in the embryo (8, 9).

Recently, several groups found roles for EG5 independent of cell division, for example, cell migration, intracellular vesicle trafficking, or regulation of primary cilia dynamics in several cell models including in postmitotic neurons (10–15). These studies could explain the pleiotropic and variable phenotype characteristic of MLC syndrome, potentially related to the diverse functions of EG5, which may even be cell-type dependent.

Here, we investigate the clinical features and disease mechanisms in patients with MLC with *KIF11* pathogenic variants, focusing on lymphatic function. They typically presented with congenital onset lower limb lymphedema, mostly in the dorsum of the foot. Clinically, their phenotype resembles that of Milroy disease, and lymphoscintigraphy scans showed functional aplasia. Significant issues with intestinal lymphangiectasia were noted in one of the individuals, which has only been reported once in the MLC patient population (16) but is characteristic of other primary lymphatic anomalies (17, 18).

We hypothesize that there is a critical role for *KIF11* (EG5) in the lymphatics, possibly through interactions with VEGFR3. We show that *KIF11* coexpresses with *VEGFR3* in the initial steps of lymphangiogenesis in early human and mouse embryonic development. In zebrafish, *kif11* expression is associated with proliferation of endothelial progenitors. In vitro loss-of-function models show that *KIF11* inactivation decreases proliferation, migration, and sprouting after VEGFC treatment of serum-starved human dermal lymphatic endothelial cells (LECs). Here, we provide insights into the association of EG5 with lymphatic development and function in health and disease. More research into the specific roles of EG5 at defined lymphatic developmental stages is required to characterize disease mechanisms and delineate target treatments for patients with MLC.

## Results

### Patients with MLC show lymphatic functional defects.

**Figure 1:**
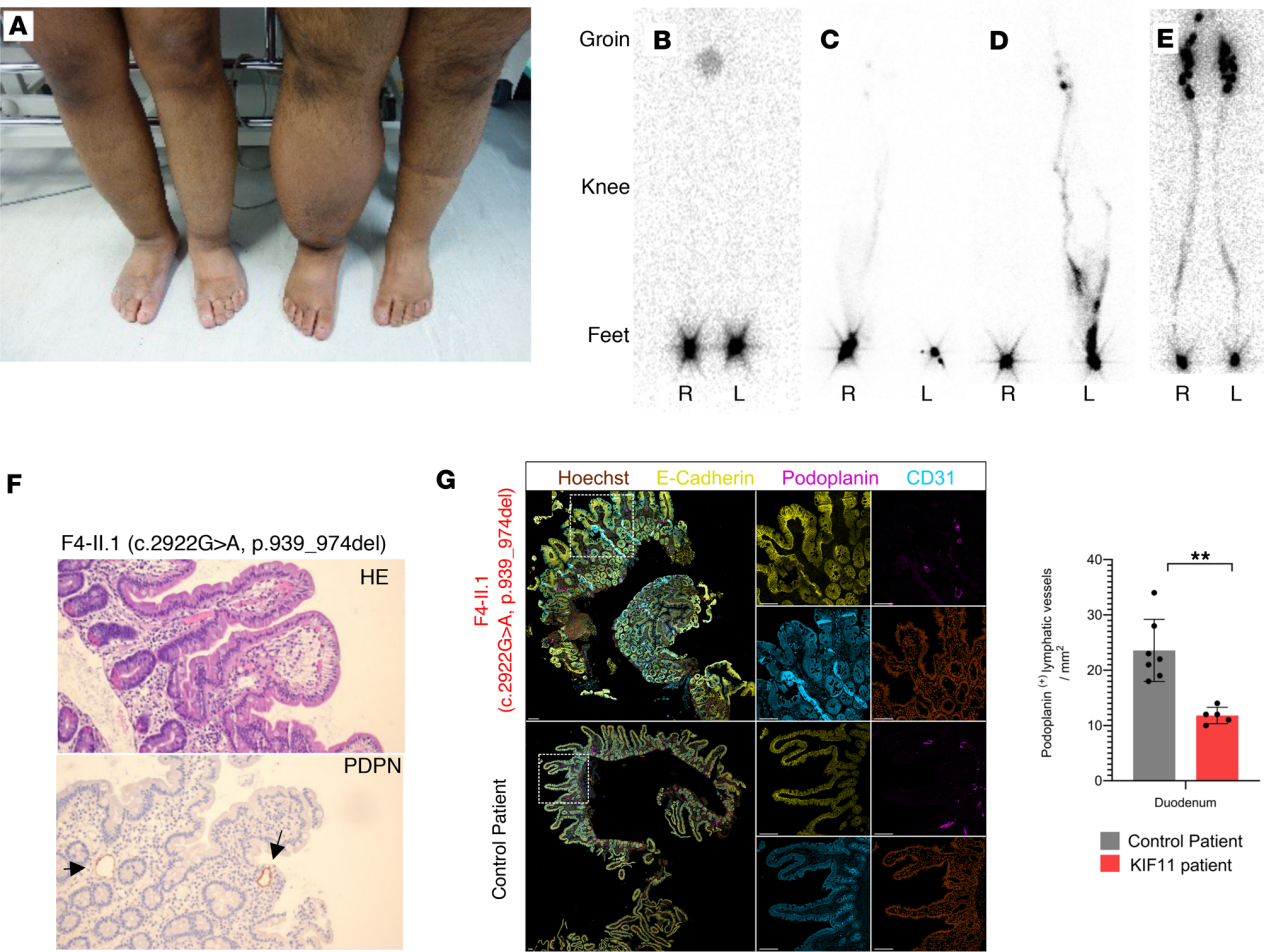
Patients with MLC show lymphatic abnormalities. (**A**) Photography of lower limbs of patients F3-II.1 and F3-II.2 showing asymmetrical lower limb lymphedema. (**B**–**E**) Lower limb lymphoscintigraphy 2 hours after injection of radionuclide (technetium-99) was used to image the lymphatic system (anterior view shown here). (**B**) Bilateral functional aplasia evidenced by no lymphatic drainage in either leg. (**C**) Significantly reduced function with rerouting in the right limb and functional aplasia of the left. (**D**) Functional aplasia of the right limb and abnormal tortuous tracts with patchy superficial rerouting on the left. (**E**) Unaffected patient with symmetrical transport of radionuclide tracer from injection sites in the feet up to the inguinal lymph nodes via main lymphatic vessels. (**F**) Intestinal biopsy of patient F4-II.1 with *KIF11* variant c.2922G>A; p.939_974del. H&E staining shows aberrant morphology of the lacteals; podoplanin (PDPN) detected slightly enlarged lymphatic vessels (arrows). (**G**) Human duodenum sections derived from same patient as in **F** and an unaffected control were subjected to immunofluorescence staining. Selected magnifications (framed areas) depict staining for Hoechst (brown; right column, second and fourth images), E-cadherin (yellow; middle column, first and third images), PDPN (magenta; right column, first and third images), CD31 (cyan; middle column, second and fourth images). Scale bars: 100 μm. Immunofluorescence staining revealed significantly reduced lymphatic vessel density compared with healthy control samples based on PDPN-positive area per mm^2^ tissue area. Two-tailed Student’s *t* test; **P < 0.01. **E** is a historical standard taken from authors’ archive: “Methods of Imaging the Lymphatic System” by City St George’s University of London licensed under CC BY-SA-4.0 (59).

Lymphedema in patients with MLC is mostly bilateral, affecting lower limbs (Figure 1A) (1, 3). We evaluated 10 patients with an MLC phenotype, 6 individuals from 4 families carrying 2 different unreported *KIF11* variants: c.2680C>T; p.(Q894*) and c.2922G>A; p.(P974=), and 4 individuals from 2 previously reported families (3) carrying pathogenic variants in *KIF11,* c.1159C>T; p.(R387*) and c.1039_1040delCT; p.(L347Efs*8). Clinical summaries are in Supplemental Tables 1 and 2 (supplemental material available online with this article; https://doi.org/10.1172/jci.insight.177656DS1). Variant summaries are in Supplemental Table 3, and pedigrees are shown in Supplemental Figure 1.

Lymphatic function of the lower limbs was assessed in 3 individuals using lymphoscintigraphy. Quantification of the tracer signal 2 hours after injection revealed that almost 100% of activity was retained in the feet of the most affected limbs with a reduced uptake in the groin of 0.1%–0.4% (Table 1). Proband F1-II.1, who presented with congenital bilateral lymphedema, showed absence of radioactive isotope uptake from the web spaces between the toes (i.e., functional aplasia) as described previously (3) (Figure 1B). Two siblings, F3-II.1 and F3-II.2, presenting with microcephaly and congenital bilateral lower limb lymphedema (Figure 1A) also demonstrated abnormalities on lymphoscintigraphy images (Figure 1, C and D). One leg was more affected than the other in both siblings but with evidence of functional aplasia in one leg; thus, the findings are in keeping with an underlying bilateral primary lymphedema compared with the control (Figure 1E, a historical standard from authors’ archive).

Proband F4-II.1 presented with dysphagia, intermittent diarrhea, and failure to thrive; a clinical observation not typical of MLC. Podoplanin staining of intestinal biopsies showed dilated lymph vessels (lacteals) in the duodenum (Figure 1F), and together with the significantly increased alpha 1 antitrypsin (A1AT) values in the stool, was consistent with protein-losing enteropathy caused by intestinal lymphangiectasia. Immunofluorescence staining revealed reduced lymphatic vessel density compared with healthy control samples based on podoplanin-positive area per tissue area (Figure 1G).

### Patients with MLC show reduced expression of KIF11 (EG5), pointing to haploinsufficiency as the disease mechanism.

**Figure 2:**
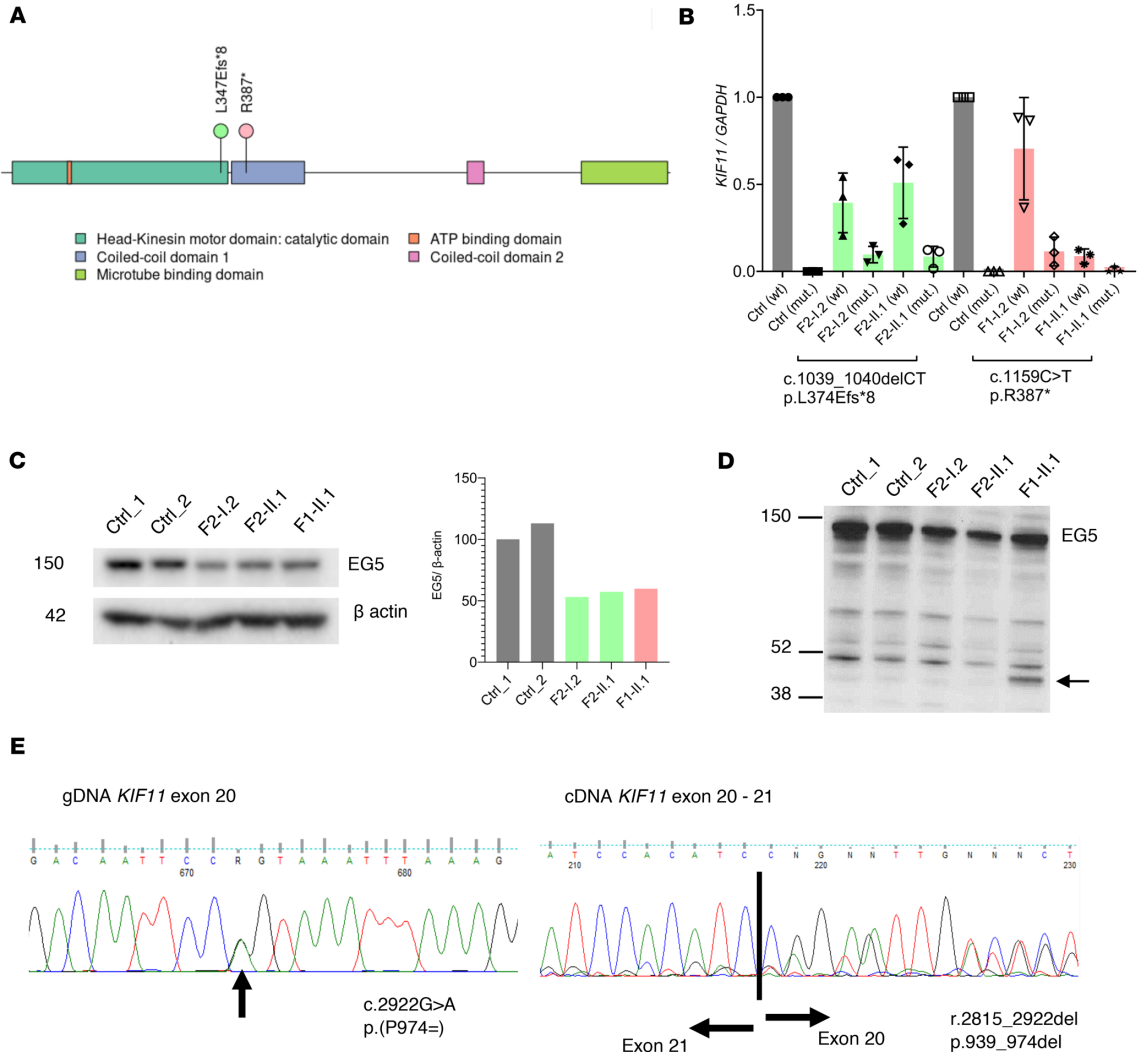
Reduced expression of *KIF11* (EG5) and splicing abnormalities suggest haploinsufficiency as the disease mechanism in MLC. (**A**) EG5 (*KIF11*) protein domain structure indicating the position of the MLC variants p.L347Efs*8 and p.R387*. (**B**) qRT-PCR analysis of blood using allele-specific primers (mut: patient variant allele) showing strong reduction but not a complete absence of the mutant mRNA in all samples (F2-I.2, F2-II.1, F1-I.2 and F1-II.1), suggesting nonsense-mediated RNA decay. WT *KIF11* mRNA levels decreased by up to 50% in patients compared with controls. Data represent average relative expression (*n* = 3 experiments) ± SD. (**C**) Western blot analysis of lymphoblastoid cell line lysates indicate approximately 50% reduction in the levels of WT EG5 protein (119 kDa) in the patient samples (F2-I.2, F2-II.1, and F1-II.1) using C-terminal binding EG5 antibody (NB500-181, Novus Biologicals). Average of 2 technical repeats of identical biological materials is shown with β-actin as loading control. (**D**) Western blot analysis with an N-terminal binding anti-EG5 antibody (CC10014, Cell Applications) showed the presence of a truncated protein of the expected size in patient F1-I.2 (arrow). The position of molecular mass markers (in kDa) is indicated on the left of the gel. (**E**) Left panel: Sanger sequencing of gDNA from proband F5-II.1. PCR product shows a compound peak in exon 20, which is the synonymous *KIF11* c.2922G>A; p.(P974=) variant. Right panel: Sanger sequencing of cDNA from F5-II.1. Black line indicates the boundary between the last base of exon 20 and first base of exon 21. Notice the heteroduplex in exon 20 indicating exon skipping. Analysis of the full trace identified a loss of the last 108 bases in exon 20 (r.2815_2922del), which is predicted to lead to a shorter EG5 protein similar to that of the synonymous variant c.2922G>T as shown by others (46).

To ascertain the disease mechanism in MLC, we analyzed the expression of *KIF11* in lymphoblastoid cell lines, blood, and saliva from 4 patients carrying pathogenic variants (leading to a premature termination) in the motor domain (L347Efs*8) and the coiled-coil domain 1 (R387*) (Figure 2A). These regions are involved in protein oligomerization (19) and vesicle tethering (20), respectively, and thus it seems likely that both variants will negatively affect EG5 function. qRT-PCR analysis of blood (Figure 2B) and saliva (data not shown) using allele-specific primers for the WT allele and each of the mutant alleles showed a strong reduction but not a complete absence of the mutant mRNA in all samples. This fits with nonsense-mediated RNA decay and the expected level of WT *KIF11* mRNA of 50% in patients’ samples compared with controls. Western blot analysis of protein lysates from lymphoblastoid cell lines also showed around 50% of EG5 protein levels compared with controls (Figure 2C). Interestingly, a truncated protein of expected size (43 kDa) was detected in F1-II.1 (Figure 2D).

### A synonymous variant in KIF11 causes cryptic splicing, which leads to exon skipping.

Three patients with MLC in this study carried the same synonymous variant c.2922G>A; p.(P974=) (Supplemental Table 1), and in silico prediction tools suggested the variant is pathogenic (Supplemental Table 3). RNA was isolated from patient F5-II.1, converted into cDNA, and Sanger sequenced. The synonymous variant caused cryptic splicing, which led to the removal of a 108 bp fragment of exon 20 (r.2815_2922del) (Figure 2E), possibly leading to a shortened EG5 protein (p.V939_P974del) lacking part of the BimC-box tail domain. This C-terminal domain appears to be a crucial region in microtubule binding, and a previously described pathogenic variant (p.R944C) in this region has been reported (3).

### KIF11 coexpresses with lymphatic VEGFR3-positive structures during human embryonic development.

**Figure 3:**
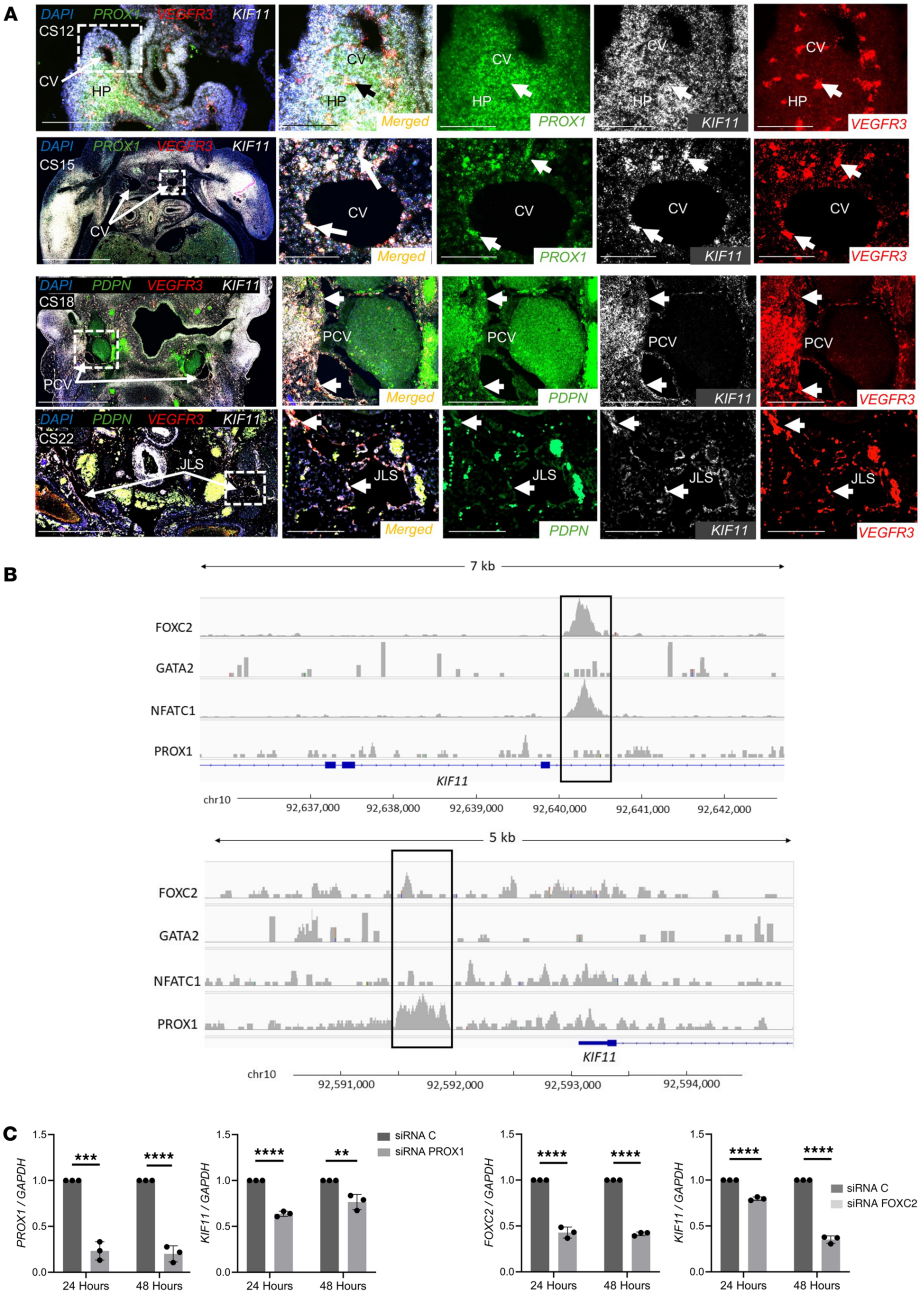
*KIF11* expression during human embryonic development and transcriptional control in adult lymphatic endothelial cells. (**A**) Human embryonic slides corresponding with different embryonic developmental Carnegie stages (CS12, CS15, CS18, and CS22) were hybridized with fluorescence probes for *KIF11* (white), *PROX1* or *PDPN* (green), and *FLT4* (red). Magnified regions marked with a square box in overview images contain the precardinal veins (PCVs), cardinal veins (CVs), and jugular lymphatic sacs (JLSs), and arrows point to regions of coexpression between *KIF11* and lymphatic endothelial marker expression. HP, hepatic primordia. Scale bar: 200 μm. (**B**) ChIP-Seq analysis in cultured human dermal LECs identified an intronic region of *KIF11* bound by FOXC2 and NFATC1 (top panel) and PROX1 binding 1.3 kb upstream of *KIF11* promoter (bottom panel). (**C**) LECs were treated with siRNA *PROX1* or siRNA *FOXC2*, and *KIF11* expression was evaluated by qPCR. Bars represent mean relative expression ± SEM from *n* = 3 experiments. **P < 0.01, ***P < 0.001, ****P < 0.0001. Two-tailed unpaired Student’s *t* test.

In silico analyses show kinesin transcripts, including *KIF11*, are enriched in the endothelial cell–expressed sequence tag pool (21), and human foreskin sections showed positive costaining of EG5 with CD31 and podoplanin, confirming the expression of EG5 in adult lymphatics. Given that MLC is characterized by early-onset lymphedema, there is a need to understand *KIF11* expression patterns during lymphatic embryonic development. Human embryonic tissues at different developmental stages ranging from human Carnegie stage 12 (CS12) to CS22 were analyzed for *KIF11* mRNA expression along with different lymphatic-specific markers (*VEGFR3* and *PROX1* or *PDPN*) using RNAscope multiplexing in situ hybridization. Although coexpression of *KIF11*, *PROX1,* and *VEGFR3* was found in the hepatic primordia, little or no coexpression of *KIF11* with other lymphatic markers was seen around the developing cardinal vein at CS12 (Figure 3A). Later, at CS15, low expression levels of *KIF11,* with coexpression to *PROX1* and *VEGFR3,* were detected around the cardinal vein. At CS18, increased expression of *KIF11* with *VEGFR3-* and *PDPN*-positive cells was found around the cardinal vein. These VEGFR3-positive cells would migrate from the cardinal vein to form the primordial thoracic duct (pTD), formerly known as the lymphatic sacs (22). Later in development, at CS22, the association between *KIF11* and lymphatic markers was maintained as the lymphatic vessel network expanded. This indicates that *KIF11* is associated with the development of lymphatic vessels at the later stages analyzed.

### KIF11 expression might be regulated by lymphatic transcription factors in human LECs.

To understand the relationship between *KIF11* expression and key transcriptional regulators of lymphatic development, we analyzed publicly available ChIP-Seq data of primary human dermal LECs (23) to identify regions on *KIF11* bound by GATA2, FOXC2, NFATC1, and PROX1. We identified binding of FOXC2 and NFATC1 in intron 17 (chromosomal position chr10:92,640,060–92,640,600) and binding of PROX1 approximately 1.3 kb upstream of the *KIF11* promoter (chr10:92,591,500–92,592,000) (Figure 3B). Both peaks overlapped with ENCODE enhancer-like signals predicted from DNase hypersensitivity, histone modification, or CT-CF binding, with data combined across cell types (24). To experimentally confirm that PROX1 and FOXC2 may regulate *KIF11* expression, LECs were treated with either siRNA *PROX1* or siRNA *FOXC2* and *KIF11* expression evaluated by RT-qPCR, showing downregulation (Figure 3C).

### Kif11 coexpresses with lymphatic Flt4-positive structures during mouse embryonic development and in adult mouse intestine.

In mice, lymphatic vessels develop at around developmental day E10.0. A subpopulation of PROX1-positive cells, so called initial LECs (iLECs), leave the cardinal vein and form the pTD (22). A second lumenized lymphatic vessel, the peripheral longitudinal lymphatic vessel (PLLV), is formed simultaneously, which represents a potential source or vascular connection site for dermal lymphatic vessels (22). We therefore investigated the expression of *Kif11* and *Flt4* in the cardinal vein, iLECs, pTD, and dermal LECs/PLLVs.

**Figure 4:**
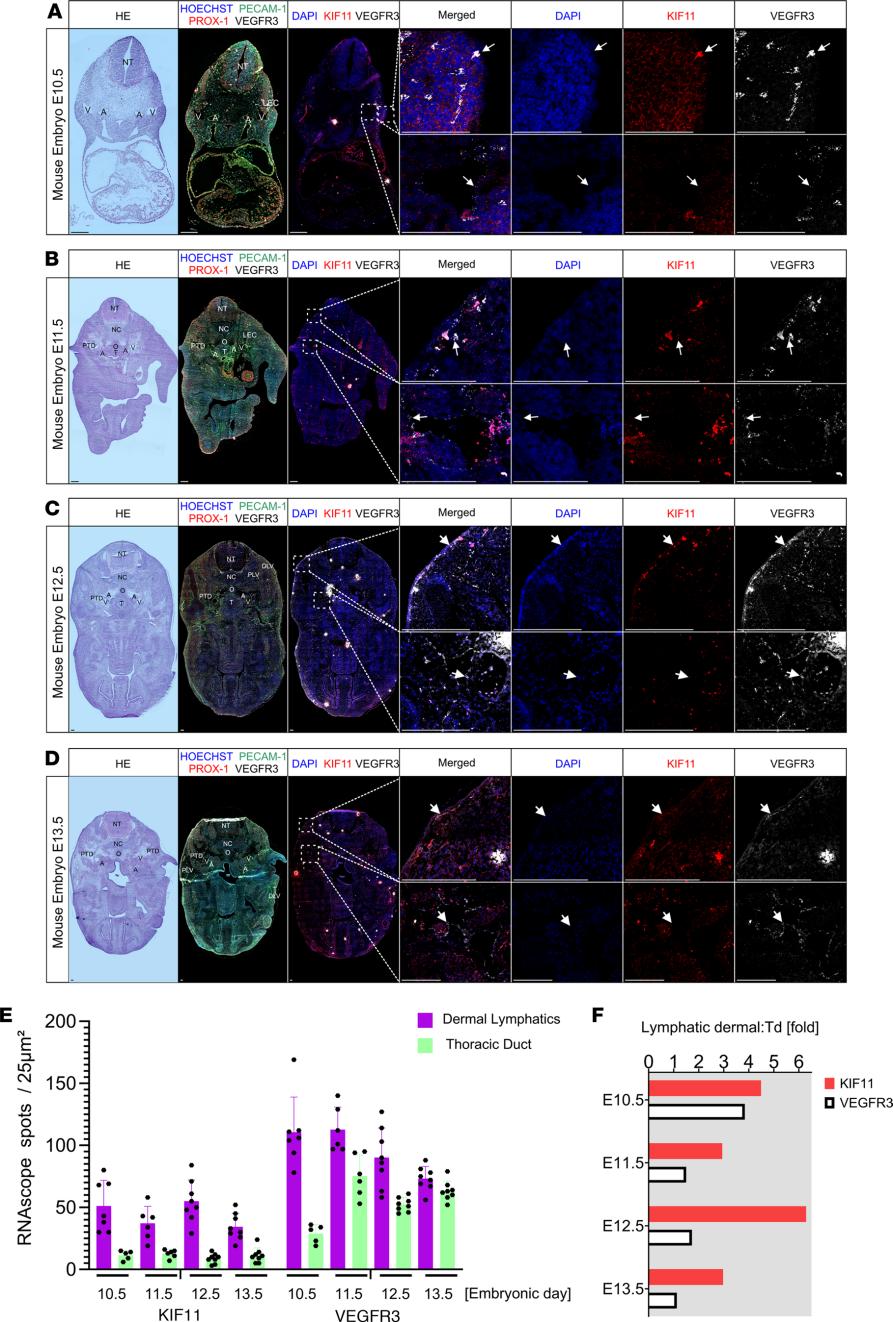
*Kif11* coexpression with *Flt4-*positive lymphatic vessels during mouse embryonic development. Mouse embryonic sections from developmental stages (**A**) E10.5, (**B**) E11.5, (**C**) E12.5 and (**D**) E13.5 were processed by H&E staining, immunofluorescence staining, or RNA hybridization. Selected magnified (framed) areas highlight embryonic regions containing dermal lymphatics and the thoracic duct. These regions were imaged using DAPI staining (blue; channel 2) as well as for *Kif11* (red; channel 3) and *Flt4* (white; channel 4) mRNA expression; merged images are also shown. Arrowheads indicate areas with coexpression of *Kif11* and *Flt4* mRNA. Additional labeling was added to facilitate the understanding of the embryonic anatomy: A, artery; DLV, dermal lymphatic vessel; LEC, lymphatic endothelial cell; NC, notochord; NT, neural tube; O, esophagus; PLV, peripheral lymphatic vessel; PTD, primordial thoracic duct; T, trachea; V, vein. Scale bars: 100 μm. (**E**) Quantification of *Kif11* and *Flt4* mRNA signals from dermal (purple) and thoracic duct (light green) of different stages of mouse embryo development. RNAscope signals were measured from 6–8 different areas of 25 μm^2^. (**F**) Bar chart represents *Kif11* (red) and *Flt4* (VEGFR3) (white) mRNA signal ratio in dermal versus thoracic duct (Td) during different stages of mouse embryo development.

First, we located the relevant lymphatic structures by H&E staining and immunofluorescence on mouse embryonic slides from E10.5 to E13.5 (Figure 4, A–D). Using RNAscope, we identified a very weak *Flt4* mRNA signal in the cardinal vein but a robust *Flt4*mRNA signal in iLECs at E10.5 (Figure 4A). From E11.5 to E13.5 when the formation of the pTD/lymph sac begins, there was an increased intensity of expression of *Flt4* in the cells lining the structure (Figure 4, B–D). This increase of signal in developing and expanding lymphatic vessels was consistent with the finding in humans at later developmental stages (e.g., CS22, Figure 3A).

*Kif11* mRNA signal was detected to coexpress with cells expressing *Flt4* from E11.5 to E13.5 in the pTD at relatively constant levels, albeit at a lower signal intensity (Figure 4E). Likewise, dermal cells with a *Flt4*-positive mRNA signal expressed *Kif11* (Figure 4, B–D). The *Kif11* mRNA signal appeared to be 3- to 5-fold higher in dermal LECs compared with that of the pTD (Figure 4F).

**Figure 5:**
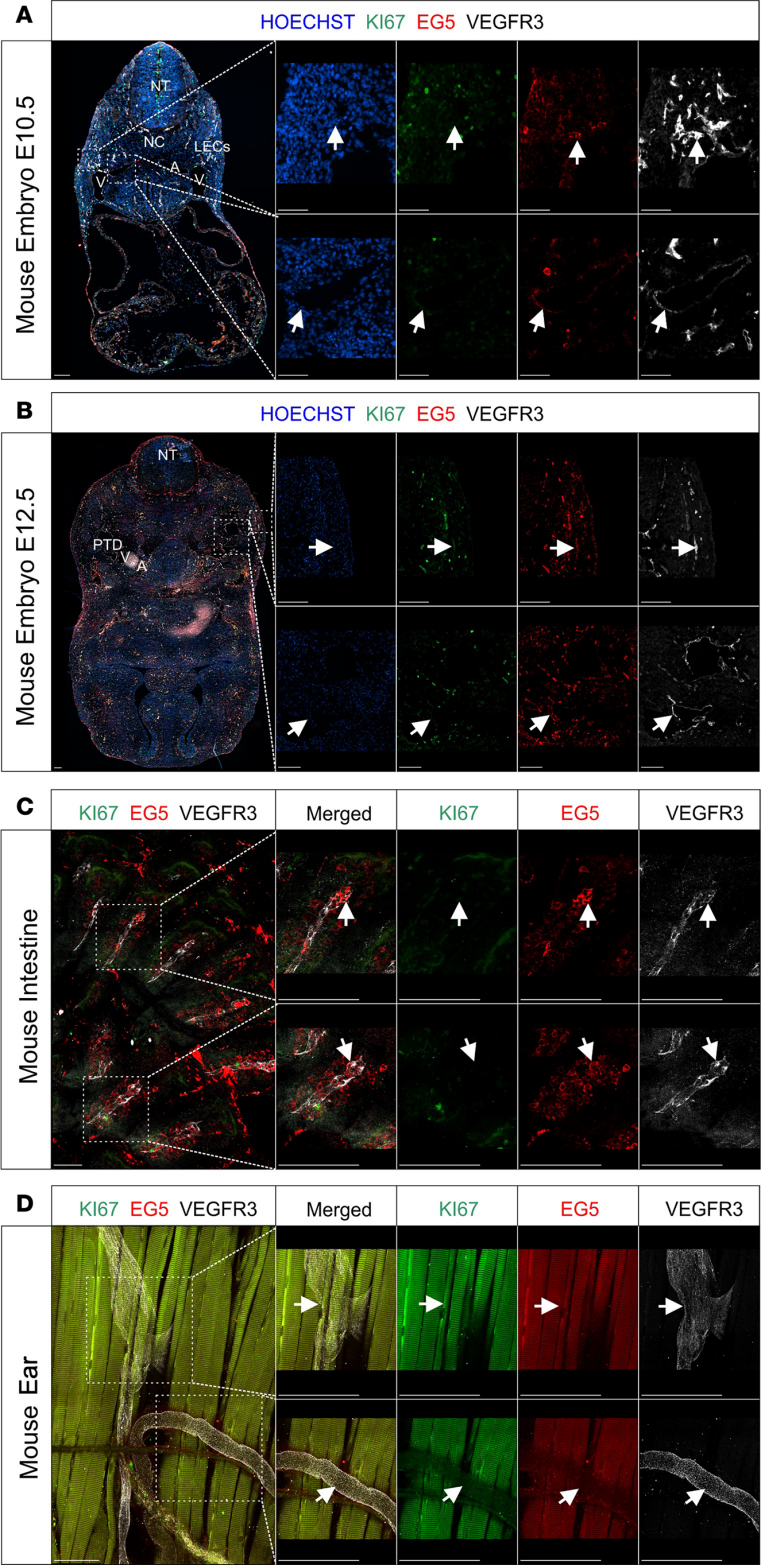
EG5 protein coexpression with VEGFR3-positive lymphatic vessels during mouse embryonic development and its expression pattern in adult mice. Mouse embryonic sections from developmental stages (**A**) E10.5 and (**B**) E12.5 were subjected to immunofluorescence staining. Selected magnified (framed) areas highlight regions containing dermal lymphatics and the thoracic duct, which were imaged for HOECHST (blue; channel 1), Ki67 (green; channel 2), EG5 (red; channel 3), and VEGFR3 (white; channel 4) protein expression. Additionally, whole-mount immunofluorescence staining was performed on (**C**) intestinal and (**D**) ear samples. Selected magnifications highlight regions with lacteals (**C**) and initial lymphatic vessels (**D**), also imaged for Ki67 (green; channel 2), EG5 (red; channel 3), and VEGFR3 (white; channel 4); merged images are also shown. Arrowheads indicate areas with coexpression of EG5 and VEGFR3. Scale bars: 100 μm. Additional labeling was added to facilitate the understanding of the embryonic anatomy: A, artery; LEC, lymphatic endothelial cell; NC, notochord; NT, neural tube; PLV, peripheral lymphatic vessel; PTD, primordial thoracic duct; V, vein.

We next performed immunofluorescence staining with an anti-EG5 antibody, anti-VEGFR3 antibody, and anti-Ki67 antibody to also assess proliferation in E10.5 and E12.5 mouse embryonic slides (Figure 5, A and B). As we had observed using RNAscope, we saw a protein coexpression pattern of EG5 and VEGFR3 that mirrored the previously detected mRNA coexpression. EG5 protein colocalized with VEGFR3-positive cells in the pTD at both the E10.5 and E12.5 stages, though the EG5 signal appeared consistently lower in intensity. Similarly, dermal VEGFR3-positive cells also expressed EG5, with stronger EG5 signal intensity in dermal LECs compared with those in the pTD. Costaining for EG5, Ki67, and VEGFR3 revealed only limited overlap, indicating a low proliferative rate during early lymphatic vessel development in the mouse at the investigated developmental stages (Figure 5, A and B).

Given the phenotype observed in patients with MLC, we performed whole-mount immunofluorescence staining in adult ear and intestinal mouse tissues isolated from 2-month-old mice. In the adult mouse intestine, we detected robust protein coexpression of EG5 and VEGFR3 in the lacteals (Figure 5C). In contrast, no detectable EG5 protein signal was observed in the adult mouse ear (Figure 5D).

To complement our mouse development investigations, we interrogated publicly available single-cell RNA-Seq (scRNA-Seq) datasets (25, 26). Analysis of single-cell transcriptomics data of healthy mouse skin tissue demonstrated *Kif11* expression was detected in proliferative LECs, with a significantly increased proportion of cells expressing *Kif11* compared with other cell types (pairwise χ^2^ *P* < 0.001, Supplemental Figure 2, A and B). This effect was also seen in an additional single-cell LEC transcriptomics dataset (Supplemental Figure 2, C and D).

### During zebrafish embryonic development, kif11 expression is associated with highly proliferative lymphatic and blood endothelial precursors.

**Figure 6:**
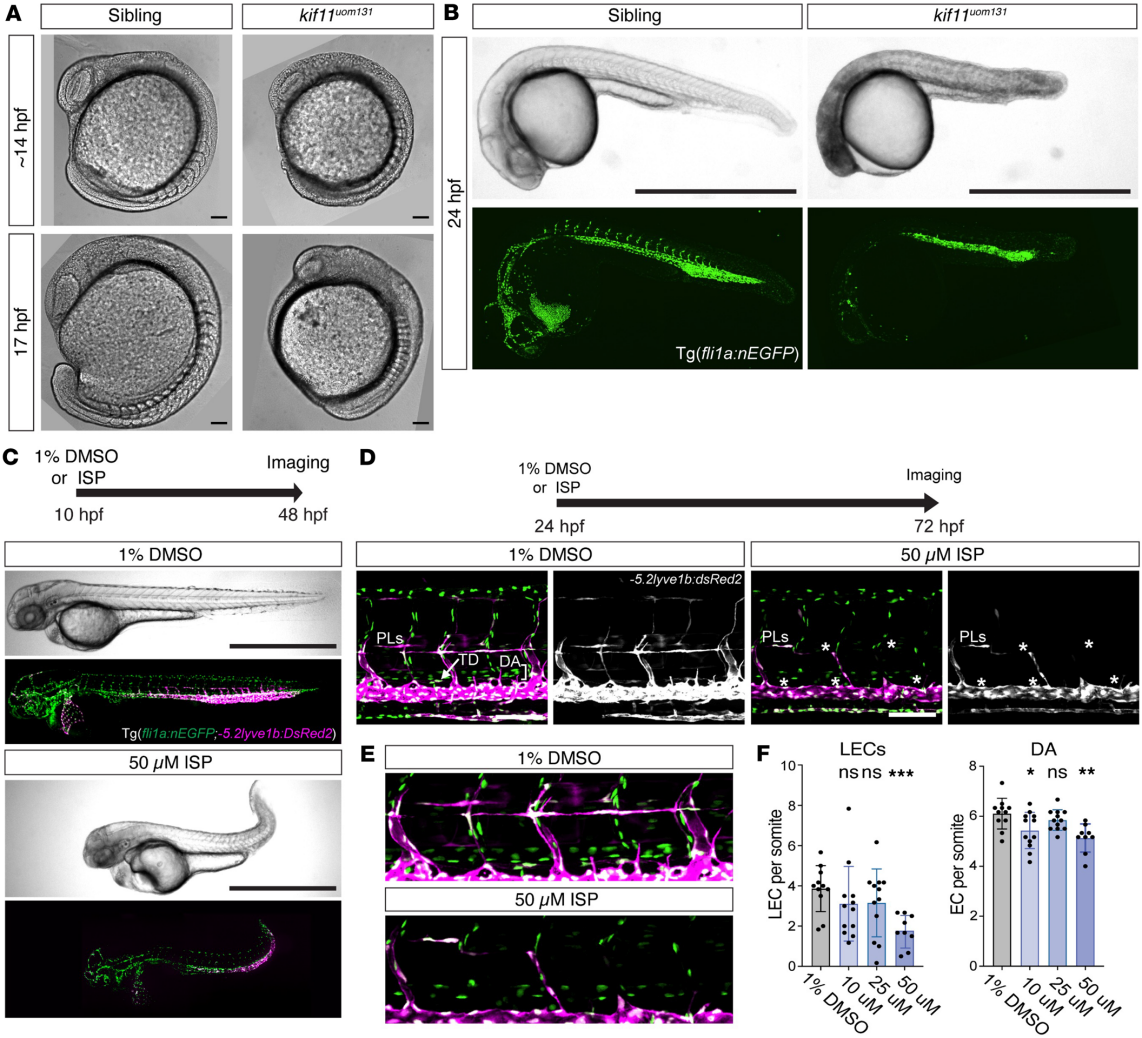
Genetic and pharmacological loss of *kif11* results in abnormal embryonic and vascular development in zebrafish. (**A**) Differential inference contrast images of 14 and 17 hpf sibling (*kif11^+/+^^or^ ^+/uom131^*) and mutant embryos (*kif11^uom131^)*. Somite defects, brain necrosis, and aberrant tail development observable in 100% of mutants at both time points (*n* = 8/8 mutants and *n* = 0/19 sibling at 14 hpf and *n* = 8/8 mutants and *n* = 0/7 sibling at 17 hpf). Scale bar: 100 μm. (**B**) Upper: brightfield images of sibling and mutant embryos at 24 hpf. Brain necrosis and reduced embryo size observable in mutants. Lower: confocal maximum projection images of Tg(*fli1a:nEGFP*) embryos showing reduced intersegmental vessel sprouting and vascular development likely consistent with delayed embryonic development (*n* = 26/26 mutants and *n* = 0/88 sibling). Scale bar: 1 mm. (**C**) Brightfield and confocal maximum projection images of DMSO control (upper) and treated (lower) embryos following exposure to 50 μM ispinesib (ISP) at 10–48 hpf. Tg(*fli1a:nEGFP;lyve1b:DsRed2)* used to visualize ECs, veins, and lymphatic progenitors (PL). ISP treatment caused brain necrosis, pericardial edema, and aberrant tail development (*n* = 26/26 ISP-treated, *n* = 27 1% DMSO). Scale bar: 1 mm. (**D**) Confocal maximum projection images of DMSO control or 50 μM ISP-treated Tg(*fli1a:nEGFP;lyve1b:DsRed2)* embryos at 72 hpf. ISP treatment 24 –72 hpf; 50 μM ISP-treated embryos showed impaired PL formation at horizontal myoseptum (quantified in **E**). Scale bar: 100 μm. (**E**) Zoomed images of DA and PLs from **D**. (**F**) Bar plots of EC numbers (per somite) in DA and PLs treated with increasing ISP concentrations (*n* = 11, 1% DMSO; *n* = 12, 10 μM ISP; *n* = 12, 25 μM ISP, *n* = 9, 50 μM ISP). Reductions in LECs and DA ECs were observed. Unpaired Student’s *t* test for normally distributed data and Mann-Whitney *U* test for non-normally distributed data. *P = 0.0261; **P = 0.0018; ***P = 0.0002; ns, nonsignificant. TD, thoracic duct; DA, dorsal aorta; EC, endothelial cell.

To model the pathogenic basis of *KIF11*-associated disease, we used CRISPR/Cas9 to generate a zebrafish *kif11* mutant model harboring an 8 bp deletion within the coding sequence (Supplemental Figure 3A). This allele was designated *kif11^uom131^*. Similar to a number of previously reported pathogenic *KIF11* variants (3, 16), the deletion induced a frameshift and subsequent stop codon, predicted to truncate the kinesin motor domain of Eg5 (Supplemental Figure 3A). Analysis of sibling (*kif11^+/+^^or^ ^+/uom131^*) and *kif11^uom131^* homozygous mutants revealed a developmental phenotype at 14 and 17 hours after fertilization (hpf). Mutant embryos displayed abnormal development of the brain and showed somitogenesis defects, as well as a delay in embryonic development based on somite numbers (Figure 6A). At 24 hpf, homozygous mutant embryos showed brain necrosis, abnormal eye development, and delayed embryonic development, including the formation of vasculature (Figure 6B). Homozygous embryos also had no circulation at 28 hpf or 48 hpf (*n* = 33/111 at 28 hpf, *n* = 11/56 at 48 hpf) (data not shown). This led to embryonic lethality by 2–3 days after fertilization (dpf), which precluded the analysis of the lymphatic lineage. Heterozygous mutants were indistinguishable from WT, including in lymphatic development (Supplemental Figure 3B). These defects are consistent with aspects of the human phenotype but could be considered more severe than the human phenotypes reported to date (1, 16). 

To further explore the impact of *kif11*/Eg5 loss of function in zebrafish, we next treated embryos with carefully optimized concentrations of the Eg5 inhibitor ispinesib (Supplemental Figure 3C). To avoid severe early developmental phenotypes, we treated at stages following embryonic gastrulation. Treatment from 10 hpf led to a partial phenocopy of the mutant phenotype described above with brain necrosis and tail extension defects (Figure 6C), suggesting that the inhibitor was likely generating a loss-of-function model. Next, we treated embryos with 50 μM ispinesib from 24 hpf and analyzed them at 72 hpf to allow normal vasculogenesis and angiogenesis to occur before assessing *kif11*/Eg5 function in vasculature. Treatment at this stage generated embryos that developed normal blood circulation and major vascular lineages, such as the dorsal aorta, precardinal veins, and intersegmental vessels; however, we observed reductions in the total number of LECs and dorsal aorta endothelial cells (Figure 6, D–F). Together, this finding suggests that *kif11*/Eg5 has a broad function in embryonic development and is important for the normal development of vasculature in the embryo.

To gain further insight into the role of *kif11*, we analyzed recently published scRNA-Seq data of WT zebrafish populations at 3 and 4 dpf (27). *kif11* expression was evaluated across all reported cell types (Supplemental Figure 3, D and F) and compared with proliferation markers *pcna* and *mki67*. *kif11* expression was positively correlated with the expression of proliferation markers *mki67* and *pcna* (Supplemental Figure 3, E and G). This suggests a role in highly proliferating endothelial cell populations that would include LECs of the developing embryo and is in line with the suggested role of this protein in cytokinesis (28).

### KIF11 inhibition impairs VEGFC-driven cell migration and sprouting in LECs.

VEGFR3 is the essential signaling protein in maintaining lymphatic function. The similarities between the congenital bilateral lower limb lymphedema and functional aplasia on lymphoscintigraphy imaging seen in Milroy disease (caused by pathogenic *VEGFR3* variants) and MLC (caused by pathogenic *KIF11* variants), along with the coexpression of *KIF11* and *VEGFR3* during human and mouse embryonic development, may suggest a common functional pathway.

With *KIF11* haploinsufficiency confirmed as the disease mechanism, we generated 2 loss-of-function approaches to block EG5 function: siRNA transfection and a specific EG5 antagonist, ispinesib, widely used to investigate EG5 function in several cell models including HUVEC (21). After 24 hours of siRNA *KIF11* transfection in LECs, EG5 protein expression was reduced by more than 80% (Supplemental Figure 4A), with the expected decrease in cell proliferation measured as the percentage of EdU^+^ LECs (Supplemental Figure 4B). Spindle pole formation after ispinesib treatment (0.5–50 nM) was evaluated by α-tubulin staining (Supplemental Figure 4D). Ispinesib at 5–50 nM caused an abnormal distribution of the filament fibers, compared with vehicle-treated cells, due to the inhibition of the kinesin motor function necessary for the assembly of the microtubules (29), verifying the inhibitory effect of ispinesib on mitotic function of EG5 at the analyzed doses. Toxicity was tested in a spheroid-based assay by measuring fluorescence uptake of a dye by viable cells. Spheroids treated with 50–100 nM ispinesib showed comparable fluorescence levels to DMSO controls and retained some ability to sprout, whereas spheroids treated with 200 nM showed increased toxicity and impaired sprouting ability (Supplemental Figure 4E).

**Figure 7:**
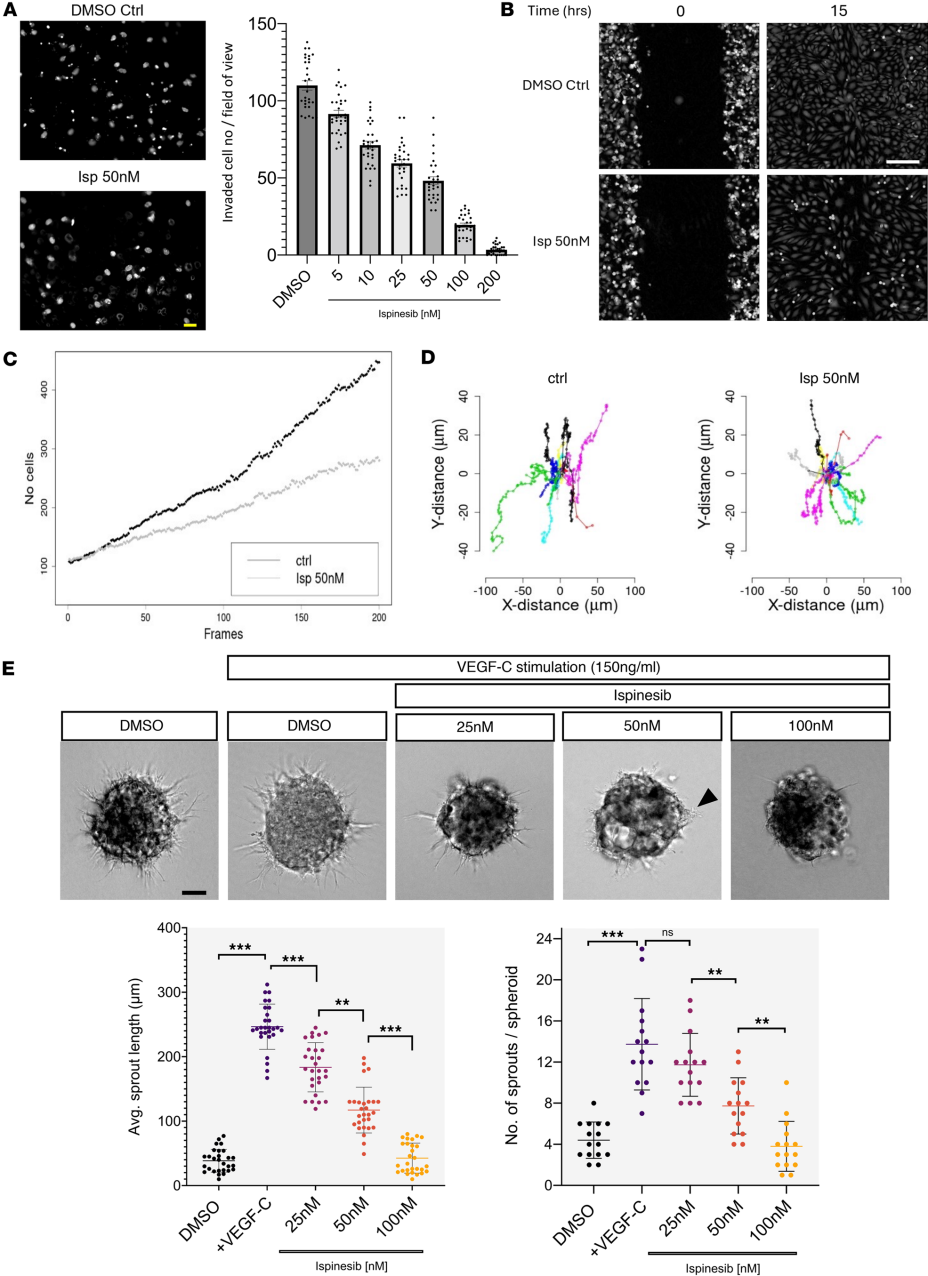
*KIF11* deficiency impairs LEC migration and sprouting. (**A**) 5 x 10^4^ serum starved cells were plated per fibronectin-coated Transwell chamber and given 10 hours to migrate in the presence of increasing concentrations of ispinesib (0 to 200 nM) before fixation and staining of the nuclei with DAPI. Resulting membranes (2 examples on left) were scored for the movement of nuclei through the Transwell membrane and results represented in a plot (right). Data are shown as SD, *n* = 3 biological replicates (with 2 technical replicates per biological replicate). Scale bar: 50 μm. (**B**) Single-cell tracking migration assay follows wound closure ability over 15 hours in DMSO (Ctrl) and 50 nM ispinesib-treated cells. Scale bar: 200 μm. (**C**) Number of cells in experimental wound-window area for Ctrl and cells treated with 50 nM ispinesib over time (frames). (**D**) Distance and direction of a subset of cells illustrated as star plots of cell tracks for Ctrl and cells treated with 50 nM ispinesib. (**B**–**D**) One representative experiment is shown from *n* = 2. (**E**) 3D spheroid sprouting assay. Sprouting was stimulated in LECs by VEGFC 150 ng/mL either in the presence of DMSO vehicle or several doses of ispinesib (25, 50, 100 nM). Average sprout length in μm (28 spheroids analyzed) and total number of sprouts per spheroid (15 spheroids analyzed) were quantified in each condition. One representative image is shown per condition from *n* = 2 biological repeats. *P < 0.05, **P < 0.01, ***P < 0.001, ns, nonsignificant. Two-tailed unpaired Student’s *t* test. Scale bar: 100 μm.

To investigate the possible role of EG5 in regulating LEC migration, we first performed in vitro Transwell migration assays to measure the ability of LECs to migrate toward VEGFC. Ispinesib inhibited migration in a dose-dependent manner, and a 50% reduction was seen after 50 nM ispinesib treatment (Figure 7A). With an approximately 50% loss of *KIF11* expression in patients with MLC (Figure 2, B and C), we sought to use 50 nM ispinesib for further experiments as our haploinsufficiency in vitro model.

To further analyze migration parameters in more detail, we performed wound healing experiments tracking single cells moving into the wound. The aim was to study the role of EG5 on cellular migration, minimizing interference from cell division, considering that the average full cell-cycle length of primary LECs is more than 20 hours. The wound was fully closed after 15 hours in the control condition, but a delay was observed in cells treated with 50 nM ispinesib (Figure 7B and Supplemental Videos 1 and 2), and a reduction of the number of cells in the experimental wound window over time was also observed (Figure 7C). Star plots showed different direction migration patterns between control and ispinesib treatments (Figure 7D), and velocity analysis indicated a significant reduction of individual cell speed in ispinesib-treated cells with the mean velocity of cells at 2.08 for the control versus 1.94 for cells treated with 50 nM ispinesib (Wilcoxon rank-sum test; *P* < 2.2 × 10^–16^).

Lymphatic sprouting is a key process driving lymphangiogenesis in the developing lymphatic system, which requires collective action of both proliferation of stalk and migration of tip cells (30). We assessed the role of EG5 in LEC sprouting using an in vitro 3D spheroid assay. After 24 hours, 150 ng/mL VEGFC increased the average sprouting length by 60% and the number of sprouts by 30% per spheroid compared with the vehicle control. This was reduced in a dose-dependent manner by ispinesib treatment (Figure 7E); 50 nM ispinesib caused approximately 50% reduction of both the average length and the number of sprouts compared with VEGFC only, and 100 nM ispinesib completely abolished VEGFC-induced sprouting. Overall, the data suggest a role for EG5 in LEC sprouting, probably due to a combination of the impact on both proliferation and migration. To differentially test the effects purely on migration, we attempted to inhibit proliferation prior to ispinesib treatment; however, in our hands, the combination of ispinesib and aphidicolin (a cell cycle inhibitor) was very damaging for the cells (data not shown).

### KIF11 inhibition reduces VEGFC-induced VEGFR3 signaling, leading to loss of AKT and MAPK phosphorylation.

**Figure 8:**
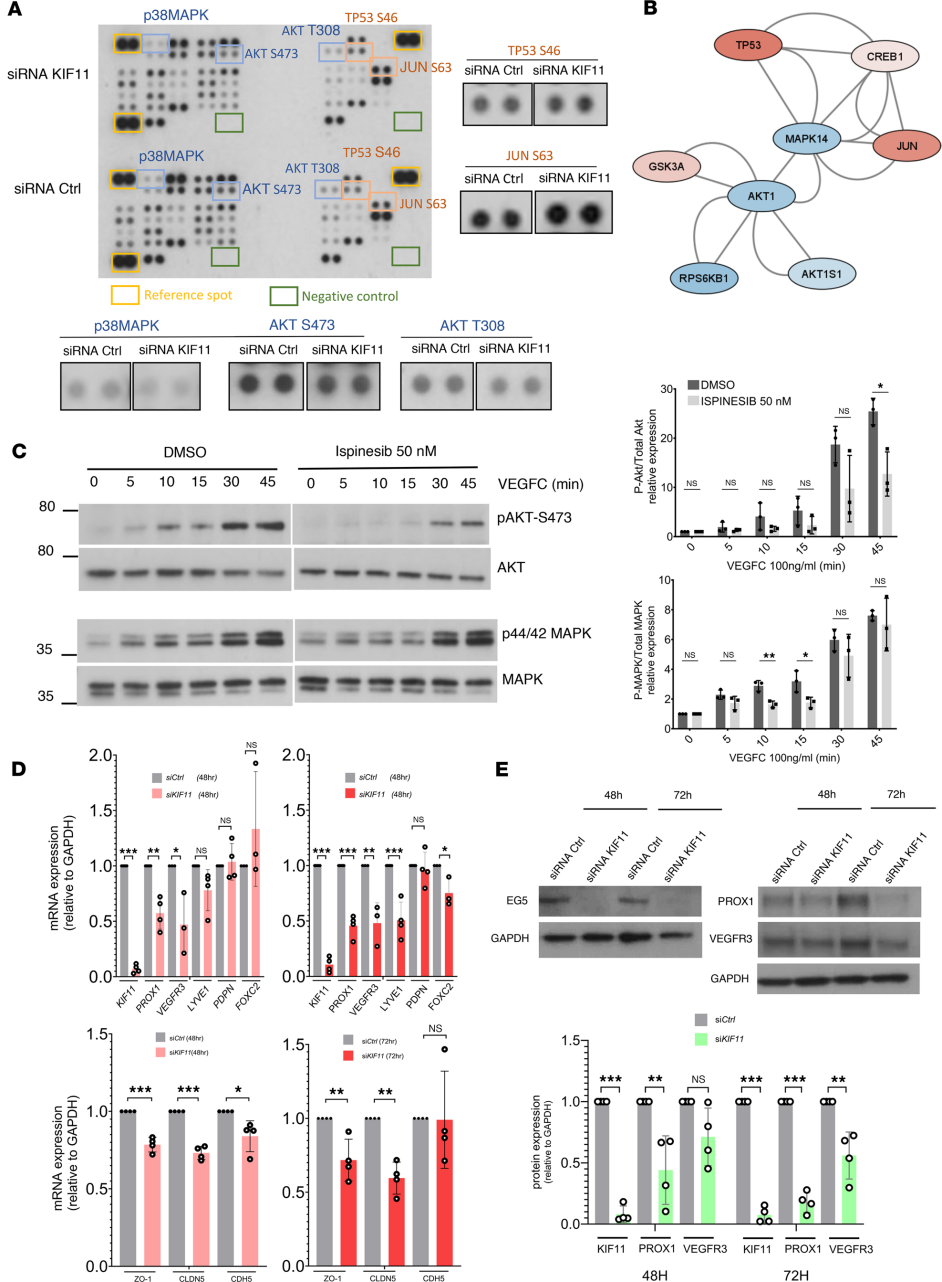
*KIF11* deficiency alters MAPK and AKT signaling pathways and reduces expression of lymphatic cell identity and cell-cell junction markers. (**A**) Human phospho-kinase array showing changes in phosphorylation levels of 43 kinases. Dots have been magnified for selected kinases with decreased (in blue) or increased (in red) phosphorylation. (**B**) Network pathway analysis using STRING and Cytoscape reveals known interactions and the directionality of the phosphorylation change in siRNA *KIF11*-treated cells (blue, decreased; red, increased). (**C**) Effect of ispinesib treatment (50 nM) on AKT and MAPK phosphorylation in LECs over time after stimulation with VEGFC (100 ng/mL). DMSO (vehicle) as control. Phosphorylation was analyzed with anti-pAKT S473 and anti-p44/42 MAPK Thr202/Tyr204 (upper panels) and levels of AKT and MAPK protein expression with anti-AKT and MAPK antibodies (lower panels). Molecular mass markers (in kDa) are indicated on the left. Blots are quantified on the right. *P < 0.05, **P < 0.01, ns, nonsignificant. Two-tailed unpaired Student’s *t* test. (**D**) *FLT4* (VEGFR3), *PROX1*, *PDPN, FOXC2*, *LYVE1, TJP1* (ZO-1)*, CLDN5*, and *CDH5* gene expression related to *GAPDH* in LECs treated with siRNA 6 *KIF11* for 48 and 72 hours analyzed by qPCR (*n* = 3–4 experiments). *P < 0.05, **P < 0.01, ***P < 0.001, ns, nonsignificant. Two-tailed unpaired Student’s *t* test. (**E**) Western blot analysis and quantification of PROX1 and VEGFR3 expression in LECs treated with siRNA *KIF11* for 48 and 72 hours. GAPDH was used as control. **P < 0.01, ***P < 0.001, ns, nonsignificant. Two-tailed unpaired Student’s *t* test. (**C**–**E**) Bars represent mean relative expression ± SEM. (**C** and **E**) One representative image is shown from *n* = 3–4 experiments.

To understand the impact of *KIF11* haploinsufficiency on LEC signaling pathways regulating migration and sprouting, we used Proteome Profiles Human Phospho-Kinase Array to detect phosphorylation changes in 43 human kinases in siRNA *KIF11*-treated LECs compared with the siRNA control. Quantification of the signal intensities demonstrated no striking changes in phosphorylation patterns, but we could detect decreased phosphorylation of p38MAPK and AKT1 (at both positions Ser-473 and Thr-308), and an increase in phosphorylation of other kinases including JUN and TP53 (Figure 8A), the last associated with spindle defects and stress response (31). We explored the molecular interactions derived from the human phospho-kinase analysis, plus the already known interactions described in the literature and public databases, by pathway analysis using STRING and Cytoscape. A selection of the kinase networks generated is shown in Figure 8B, showing interactions and the direction of the phosphorylation changes (blue, decrease of phosphorylation; red, increase of phosphorylation).

We focused our attention on the AKT and MAPK signaling pathways that appeared downregulated after siRNA *KIF11* treatment, which are 2 of the major downstream effectors of tyrosine kinase receptor signaling including VEGFR3. LECs were pretreated overnight with DMSO (vehicle) or 50 nM ispinesib and were stimulated the next day with 100 ng/mL VEGFC for up to 45 minutes. VEGFC treatment activated the phosphorylation levels of MAPK and AKT both in DMSO- and ispinesib-treated cells. Ispinesib treatment resulted in a significant reduction of MAPK phosphorylation at 10–15 minutes and AKT phosphorylation at 45 minutes after VEGFC stimulation compared with DMSO treatment (Figure 8C).

### KIF11 knockdown downregulated PROX1, VEGFR3, and cell junction molecule expression.

PROX1 is a master regulator of LEC specification (32), regulating genes and proteins important for maintenance of LEC identity such as VEGFR3 and podoplanin (33, 34) and transcription factors essential for lymphatic valve morphogenesis like FOXC2 (35). To determine whether downregulation of lymphatic markers might also contribute to the lymphatic defects observed with *KIF11* haploinsufficiency, we transfected siRNA *KIF11* for 48 and 72 hours in LECs and investigated the effect on the expression of well-characterized LEC markers. After 48 hours, we detected approximately 50% downregulation of *FLT4* (VEGFR3) gene expression and *PROX1* both at mRNA (Figure 8D) and protein (Figure 8E) levels. Other lymphatic identity markers such as *FOXC2* and *LYVE1* showed significant reduction after 72 hours, whereas the gene expression of *PDPN* remained unaffected (Figure 8D). These results were confirmed with a second siRNA sequence (siRNA KIF11 7) (Supplemental Figure 4, A and C).

Lymphoscintigraphy (Figure 1, B–D) revealed a lack of interstitial fluid uptake in patients with MLC, suggesting the initial lymphatic capillaries may be dysfunctional. The permeability of the lymphatic capillaries is critical for functional fluid uptake and is controlled by specialized junctions (36), and the importance of this specialized junctional organization has already been probed in several animal models (37–39). Since it is crucial to understand the role of EG5 in controlling lymphatic junction architecture and permeability and its contribution to lymphatic (dys)function (e.g., failure of fluid uptake in lymphatic capillaries), we looked next at the impact of *KIF11* depletion on the expression of cell junction molecules and found downregulation of *TJP1* (encoding ZO-1), *CLDN5* (encoding claudin-5), and *CDH5* (encoding VE-cadherin) (Figure 8D and Supplemental Figure 4C).

## Discussion

Autosomal dominant pathogenic variants in *KIF11* (EG5) are causative of MLC syndrome, a condition that presents with a variable spectrum of CNS, lymphatic, renal, and ocular developmental anomalies (1, 5, 16). We have a better understanding of the ubiquitous functions of EG5 and its involvement in the onset and progression of several pathologies (40). Since the association of *KIF11* with MLC (3), we have aimed to understand how EG5 variants account for the lymphatic phenotype presented in this pleiotropic disease. Very little was previously understood about the underlying mechanisms leading to lymphatic failure, partly due to the limited knowledge about the role of *KIF11* in the development and maintenance of the lymphatic system. A better understanding of the signaling pathways and molecules that regulate lymphatic development and physiology in the context of *KIF11* pathogenesis is essential to enable the development of more targeted and accurate treatments for patients with MLC.

In this study, we examined the disease mechanism(s) causing lymphedema in patients with pathogenic variants in *KIF11* and investigated the molecular and cellular interactions by which *KIF11* may regulate lymphangiogenesis. We have attempted this through (a) investigations on the clinical characteristics and lymphatic function of patients with MLC, by lymphoscintigraphy and immunohistochemistry of intestinal biopsy; (b) the use of patient-derived cells to study the expression levels of *KIF11*; (c) the analysis of *KIF11* expression during embryonic lymphatic development in mice and humans, and in adult mouse tissues; (d) the generation of MLC zebrafish models; and (e) an examination of the impact of *KIF11* haploinsufficiency on VEGFR3 signaling and lymphatic identity using primary LECs as an in vitro model.

As previously reported, lymphedema in patients with MLC is mostly bilateral, affecting the lower limbs. To determine whether the fault in the lymphatic function is at the initial lymphatic capillaries or at the collector level, we performed lymphoscintigraphy in 3 patients with MLC carrying 2 stop-gain pathogenic variants in *KIF11*. Images showed a typical functional aplasia mechanism where the tracer (or most of it) was retained at the injection points, meaning a failure or reduction of the ability of the initial lymphatics. This pattern is similar to that observed in patients with Milroy disease with pathogenic variants in *VEGFR3* (41) and suggests that the fault is in the initial lymphatic capillaries.

One patient presented with intestinal lymphangiectasia, and the staining of an intestinal biopsy revealed reduced podoplanin-positive lymphatic vessels. A reduced number of dermal lymphatic vessels was also observed in skin biopsies from patients with Milroy disease (42). Some mechanisms suggested to cause lymphangiectasia and impaired lacteal function include defective LEC polarity, valve maldevelopment, lymphatic vasculature hyperplasia, and junctional destabilization (43, 44). Given the critical role of the lacteals in fat absorption and digestion (45), the lymphangiectasia observed is likely to explain the intestinal problems in this patient. Further analysis of several blood and lymphatic markers together with junctional proteins could reveal additional structural and/or functional alterations. Only one case of intestinal lymphangiectasia had been observed in MLC before (16); therefore, our finding confirms that intestinal lymphangiectasia should be considered a feature of MLC, albeit not typical, and clinicians should perform investigations accordingly. This finding also provides new suggestions for clinicians for patient care (e.g., prescription of a low-fat diet for intestinal lymphangiectasia in patients with MLC).

Scientists have searched for the molecular disease mechanisms of MLC, suggesting pathogenic variants would most likely result in loss of function of *KIF11* due to nonsense-mediated mRNA decay, premature truncation of the protein, or splicing abnormalities (16). In this study, we looked at the impact of *KIF11* variants on mRNA and protein expression levels in lymphoblastoid-transformed cells isolated from the blood of patients (together with blood and saliva samples), confirming that the 2 stop-codon variants investigated resulted in a reduction but not total loss of the mutated mRNA. As expected, qPCR and Western blot analysis revealed an approximately 50% reduction of *KIF11* expression, and in one case a shorter truncated EG5 protein was detected. A synonymous variant in *KIF11* investigated in this study caused cryptic splicing, which led to exon skipping predicted to lead to a shorter EG5 protein. Others showed a similar effect for the synonymous variant c.2922G>T (46). Therefore, we conclude that *KIF11* haploinsufficiency is the likely underlying key disease mechanism in MLC.

We believe that to be able to explain the pathophysiology of MLC, it is necessary to understand the association of *KIF11* with the development of the lymphatic system, particularly since the expression pattern of *KIF11* in the lymphatics during development and adulthood has been poorly investigated. The early onset of lymphedema in MLC also suggests that the interaction occurs early in development. We found *KIF11* expression was associated with the development of lymphatic structures both in human and mouse embryos using RNAscope in situ hybridization. These findings were also confirmed by immunofluorescence of mouse embryos. Coexpression of *KIF11* was detected together with lymphatic endothelial markers such as PROX1, VEGFR3, or podoplanin. Interestingly, we found an association of *KIF11* regulatory regions with the lymphatic transcription factors PROX1, FOXC2, and NFATC1. Another intriguing finding was to discover that the *Kif11* mRNA signal was higher in dermal LECs compared with pTD/lymph sacs, which could indicate a distinct role for *KIF11* (EG5) in discrete lymphatic vessel beds. We could speculate that the relative prominence of *Kif11* expression in dermal LECs could relate to the functional aplasia of the initial dermal lymphatics observed in lymph scans from patients with MLC. Moreover, when analyzing adult mice, EG5 and VEGFR3 coexpression was detected in intestinal tissue, suggesting a role for EG5 in this tissue after embryonic development that could explain intestinal complications in patients with MLC postnatally.

Heterozygous mutant mice generated with a gene-trap insertion in *Eg5* appear phenotypically normal. In contrast, embryos homozygous for the *Eg5*-null allele display signs of a proliferation defect, suggesting EG5 is essential for cell division during early development (9). In another study, Chauvière et al. demonstrated that heterozygous mice are healthy, fertile, and show no detectable phenotype, whereas *Eg5*^–/–^ embryos die during early embryogenesis (8). None of these studies attempted to look at the impact of *Kif11* deficiency in the lymphatic system.

The generation of in vivo models to further investigate *KIF11*’s role in lymphatic function has proved a difficult journey, probably because of the critical role of EG5 in mitosis and the need for depleting *KIF11* early during embryo development to study its role in lymphatic development. Analysis of scRNA-Seq data of WT zebrafish cell populations at different developmental stages confirmed the expression of *kif11* was associated with highly proliferative lymphatic and blood endothelial precursors during zebrafish embryonic development. This could explain why we observed severe defects in cell survival and development that lead to early embryonic lethality. Therefore, even with the introduction of a deletion mimicking the variants observed in some patients with MLC, we do not see a comparable phenotype in zebrafish. It is possible that *kif11* function may be compensated by another kinesin motor protein or that divergence in kinesin motor protein functions is at play in zebrafish. A second *kif11* loss-of-function zebrafish model was developed by treating the embryos with ispinesib that caused a reduction in the total number of LECs and dorsal aorta endothelial cells during the development of the vasculature. Altogether, this work demonstrates an essential role in early development for *kif11* and its requirement for embryo survival and vascular development in zebrafish.

We then turned to in vitro models to investigate the possible role of *KIF11* in LECs. We focused on cell migration since the coexpression of *KIF11* and *VEGFR3* mRNA in the primordial lymphatic developing sacs could point to a possible participation of EG5 in the proliferative and migratory activity of the LEC precursor cells leaving the cardinal vein. Moreover, previous publications have highlighted this non-mitotic role linking EG5 with migration in several cell types (11, 47, 48). Our experiments suggest that LEC migration is affected by EG5 inhibition, which could be explained by the importance of microtubules in cell locomotion. Studies show how suppression of microtubule dynamics restrained forward progression and impaired directionality (49) and is dependent on the right level of kinesin expression (50).

VEGFR3 is the master regulator of lymphatic function and development, and we have previously shown that patients with Milroy disease present with reduced size of lymphatic capillaries in their skin (42). We have also observed reduced podoplanin-positive structures in the intestinal biopsy of a patient with MLC. The remarkable similarities between the bilateral congenital pedal lymphedema seen in Milroy disease (caused by pathogenic *FLT4* variants) and MLC (caused by pathogenic *KIF11* variants) point to a plausible convergence of both molecules in the same functional signaling axis. The coexpression of *KIF11-* and *FLT4-*positive vessels during human embryonic development supports this hypothesis of a common functional pathway.

We have observed a downregulation of AKT and MAPK phosphorylation after EG5 inhibition. Further research will be needed to investigate the specificity of VEGFC on the observed effect on AKT and MAPK phosphorylation after EG5 inhibition. A decrease of VEGFR3 signaling through loss of AKT activation could have a detrimental effect on the migratory behavior of LECs, as it has been shown that hyperactivation of PIK3CA confers a migratory LEC phenotype through increasing P-AKTSer473 (51). This finding opens a new avenue for future studies to investigate the role of EG5 in the PIK3/AKT pathway and the possibility of repurposing AKT activators (e.g., SC-79) as a potential treatment of MLC (52).

*KIF11* knockdown downregulated *PROX1*, *VEGFR3*, and cell junction gene expression. Other genes associated with primary lymphatic anomalies have been shown to regulate cellular junctions and permeability. For instance, abnormal cell junctions have been associated with RASopathy cases (53, 54) and EphrinB2/EPHB4-defective mice show impaired junctions (37), which could explain the edema observed. Interestingly, other kinesins have been shown to mediate VEGFR2 cell membrane recycling through Rab11, regulating vascular permeability (55). Analysis of skin biopsies from patients with MLC could confirm junctional loss of integrity as the disease mechanism and possibly shed light on the functional aplasia observed.

In conclusion, we confirm *KIF11* haploinsufficiency in patients with MLC as the disease mechanism, and that EG5 could already play a role during embryonic lymphangiogenesis as its inhibition impairs LEC functions. We speculate whether the lymphatic impairment resulting in functional aplasia (and intestinal lymphangiectasia) observed in patients with MLC could be a consequence of dysregulated VEGFR3 signaling. This would affect the control of cell communication and signaling cascades orchestrating cell proliferation and migration during lymphangiogenesis. Our data provide insights into the study of *KIF11*(EG5) in lymphatic function, and future studies should elucidate the specific disease mechanisms connecting *KIF11* pathogenic variants and lymphedema and the possibility of exploring AKT activators as a potential treatment for patients with MLC.

## Methods

### Sex as a biological variable.

In the zebrafish experiments, sex was not considered as a biological variable since only larval stages were used (not yet having undergone sexual determination). In the mouse experiments, we examined male and female animals, and similar findings are reported for both sexes. For embryonic tissue samples, no determination of sexes was possible. For human participants, MLC affects patients from different genetic ancestries and men and women equally. Here, we present 7 male and 3 female patients of European and Asian background.

### KIF11 variants and in silico analysis.

All the relative genomic and protein positions of *KIF11*/EG5 reported here correspond to the KIF11 NM_004523.4 transcript (ENST00000260731.5) and P52732 Uniprot protein accession ID. The genomic coordinates reported refer to the GRCh38/hg38 human genome reference (Supplemental Table 3). The pathogenicity was predicted by the Combined Annotation Dependent Depletion tool (56) and allele frequency checked in gnomAD databases (57). Variants were validated in accordance with American College of Medical Genetics and Genomics and Association for Clinical Genomic Science best practice guidelines (58).

### DNA and RNA/cDNA analysis of the splice variant.

To analyze the c.2922G>A variant reported in 3 of the index cases, peripheral blood was obtained from individual F5-II.1. DNA was extracted using a standard chloroform ethanol procedure. Sequencing of DNA was performed using *KIF11* (NM_004523.4) forward primer 5′-caggtagcaagactgatcctca-3′ and reverse primer 5′-cggggtaagattgaggggta-3′ covering exon 20. RNA was collected using PAXgene Blood RNA tubes (PreAnalytiX), and total RNA was extracted using the PAXgene Blood RNA Kit (PreAnalytiX). DNAse treatment was performed to eliminate genomic DNA and cDNA synthesized from the RNA with a SuperScript II Reverse Transcriptase using about 400 ng RNA and 50 ng random primers (Invitrogen). Sequencing of cDNA was performed using primers that span exons 19 to 22 of *KIF11* (NM_004523.4): Forward 5′-GGCAGCTCATGAGAAACAGC-3′ and Reverse 5′-GCGAGCCCAGATCAACCTTT-3′. All PCR products were sequenced using BigDye Terminator v3.1 chemistry (Life Technologies) and an ABI3130xla Genetic Analyzer (Life Technologies). Sequencing traces were visually inspected in FinchTV v1.4 (Geospiza). The protein prediction tool Expasy (www.expasy.org) was used to confirm the effect of the splice-site variant on the protein sequence.

### Generation of lymphoblastoid cell lines from patients with MLC and analysis of KIF11 (EG5) expression levels in blood and lymphoblastoid cell lines.

Four patients from families F1 and F2 with MLC presenting with congenital bilateral pedal lymphedema and 2 controls were selected for the generation of lymphoblastoid cells to analyze *KIF11* expression. Epstein-Barr virus transformation was used for the generation of lymphoblastoid cell lines from the patients’ peripheral blood lymphocytes at Culture Collections, Public Health England, Porton Down. For the analysis of *KIF11* expression levels, qPCR was performed in total RNA samples extracted from blood and saliva. Extraction of RNA from blood samples was performed following the instructions of the PAXgene Blood RNA kit (PreAnalytiX, 762154). Samples were eluted in 80 μL of elution buffer provided by the kit and stored at –80°C until analysis. Extraction of RNA from saliva samples was done following the Oragene (OGR-500) collection protocol followed by the QIAGEN microkit RNA kit. RNA quality and concentration were assessed using Nanodrop. All RNA obtained was reverse transcribed using oligo dT (SuperScript III First-Strand Synthesis System, 18080-051, Invitrogen). Gene expression levels were analyzed using Platinum SYBR Green qPCR Super Mix-UDG with ROX reagent (QIAGEN) and a 7900 HT Fast Real-Time PCR System thermocycler (Applied Biosystems) following the manufacturer’s instructions. Relative gene expression levels were normalized to GAPDH. Specific primers designed for each of the variants (underlined bases showing nucleotide changes) plus primers for WT and mutant alleles were as follows: L347Efs*8: CTCTCAATCTTGAGGAAACTC/CTCTCAATCTTGAGGAAACTG/CTGATTCACTTCAGGCTTATTC; R387*: CGGGCTGCAGCAAGATCTCG/CGGGCTGCAGCAAGATCTCA/CTGAAGTGAATCAGAAACTC.

Validation of the qPCR was performed using cDNA in which the R387* variant was introduced by site-directed mutagenesis with the QuikChange XL site-directed mutagenesis kit (Stratagene, 200517). Sequences were verified by Sanger sequencing analysis. Lymphoblastoid cells lines were cultured in suspension in RPMI 1640 supplemented with 2 mM glutamine and 20% FBS. Two independent Epstein-Barr B cell lines were used as a control (gift from Nancy Hogg, Cancer Research UK London Research Institute, now part of the Francis Crick Institute, London, UK). Eg5 monoclonal antibodies against the N-terminal (CC10014, Cell Applications or NB500-181, Novus Biologicals) were used in the Western blots, and β-actin was used as an internal control.

### Isolation of intestinal biopsy from a patient with MLC and analysis of lymphatic endothelial markers.

A patient with MLC presenting with failure to thrive, dysphagia, and intermittent diarrhea and vomiting received endoscopy and had intestinal biopsies taken for analysis by the pathology department at his local hospital. The intestinal biopsy, H&E, and immunohistochemistry images were a gift from Femke Piersma (Rems-Murr Kliniken, Winnenden, Germany) and Wolfgang Tränkenschuh (Robert Bosch Klinikum, Stuttgart, Germany). Subsequent immunofluorescence staining for epithelial and endothelial markers on 5 μm tissue sections was performed according to the standard immunofluorescence histology protocol. Briefly, fixed tissue sections were washed and blocked (10% chicken serum, 0.3% Triton X-100 in PBS). Following blocking, tissue sections were incubated for 1 hour with primary antibodies (diluted in 1% BSA, 1% chicken serum, 0.3% Triton X-100 in PBS), washed 3 times in PBS-T (0.1% Tween20 in PBS), and finally incubated in Alexa dye–conjugated secondary antibodies (Life Technologies). Antibodies included rabbit monoclonal IgG anti-human E-Cadherin (EP700Y, Cell Marque); mouse monoclonal IgG anti-human Podoplanin (D2-40, Cell Marque); alpaca polyclonal VHH anti-human CD31conjugated with Alexa Fluor 647 (unpublished); donkey polyclonal anti rabbit IgG Alexa Fluor 488 (A21206, Invitrogen); and donkey polyclonal anti mouse IgG Alexa Fluor 568 (A21202, Invitrogen). After sample mounting in Mowiol (Calbiochem, 475904), samples were imaged using a Zeiss LSM 980 confocal microscope (25× oil, NA = 0.8).

### RNAscope in situ hybridization in human fetal tissue.

The human tissue was collected at developmental stages ranging from CS12 to CS22. Tissues were fixed in 10% PFA overnight at room temperature, and then fixed in Methacarn before they were processed using automated Excelsior AS (Thermo Fisher Scientific, A823-1005) to generate FFPE blocks. Sections were cut from the blocks at 8 μm using a manual rotary microtome (Leica, RM2235). Simultaneous RNA in situ hybridization was carried out using RNAscope Multiplex Fluorescent Reagent kit v2 (Advanced Cell Diagnostics, 323100 and 323270) following the manufacturer’s protocol for FFPE tissue mounted on slides, with pretreatment steps of hydrogen peroxide and protease reagents (Advanced Cell Diagnostics, 322381) and target retrieval reagents for 20 minutes at 95°C (Advanced Cell Diagnostics, 322000). The following RNAscope probes were designed and purchased from Advanced Cell Diagnostics: Hs-PDPN (catalog 539751), Hs-PROX1 (catalog 530241), VEGFR3 (Hs-FLT4; 552441-C2), and Hs-KIF11 (562641-C3). The probes were fluorescently labeled using OPAL 520 (PerkinElmer, FP1487001KT), OPAL 570 (PerkinElmer, FP1488001KT), and OPAL 650 dyes (PerkinElmer, FP1496001KT) for direct visualization under automated laser-scanning microscopy (Zeiss CellDiscoverer7).

### Mouse embryo collection, mouse tissue collection, histology, immunofluorescence, and RNAscope in situ hybridization.

See supplemental materials and methods for details.

### Zebrafish husbandry, genome editing, ispinesib treatment, imaging, and quantification.

See supplemental materials and methods for details.

### LEC culture, siRNA transfection, and ispinesib treatment.

Human dermal LECs (HDLECs) (PromoCell, C-12217 or C-12216) were cultured and maintained in endothelial cell growth medium MV2 (PromoCell, C-22022) with FCS-based supplement mix (C-39226) and recombinant human VEGFC 50 ng/mL (R&D Systems, 2179-VC-025). HDLEC single-donor lots used in this study were as follows: lot 481Z001.2 (female), lot 477Z011, and lot 470Z021.2. Transfection of cells with siRNA *KIF11* 6 (NM_004523, SI02653693, target sequence 5′-ACGGAGGAGATAGAACGTTTA-3′), siRNA *KIF11* 7 (NM_004523, SI026533770, target sequence 5′-GCCGATAAGATAGAAGATCAA-3′), siRNA *PROX1* 8 (NM_002763, SI04372998, target sequence 5′-GACCTACTTCTCCGACGTAAA-3′), siRNA *FOXC2* 7 (NM_005251, SI03074834, target sequence 5′-CCAGAACGCGCCCGAGAAGAA-3′), or siRNA All Starts negative control (QIAGEN, 1027281) was performed with Lipofectamine 2000 (Life Technologies) following the manufacturer’s recommendations. Ispinesib (Tocris), a specific EG5 antagonist, was used at various concentrations and exposure times depending on the nature of the experiment. DMSO was used as vehicle.

### LEC functional assays.

LECs were used in various functional assays such as Transwell migration, wound healing, single-cell migration tracking, and spheroid sprouting assays. See supplemental materials and methods for details.

### Statistics.

GraphPad Prism 9.0 was used for all statistical assessments apart from the single-cell migration tracking done with R. Statistical significance between 2 groups was assessed by unpaired parametric 2-tailed *t* test. Statistically analyzed data are presented as mean ± SEM or mean ± SD as specified for each analysis (described in the figure legends). Differences were considered significant when *P* was less than 0.05.

### Study approval.

Six index cases and affected family members with variants in *KIF11* were included in the study. Two were previously described (3) and 4 were direct referrals from clinicians. For the latter cases, *KIF11* variants were detected by exome sequencing in the respective molecular genetics services and by an inheritance check if parental DNA was available. Ethical approval was given by the South West London Research Ethics Committee (REC Ref: 05/Q0803/257 and 14/LO/0753) or by the Charité Ethics committee (EA4/214/19 Molekulare und Moprhpogenetische Mechanismen der Lymphödementwicklung), and written informed consent was obtained from all participants.

Human embryonic tissue was obtained from the Medical Research Council (MRC)/Wellcome Trust–funded Human Developmental Biology Resource (HDBR, http://www.hdbr.org), with appropriate maternal written consent and approval from the Newcastle and North Tyneside NHS Health Authority Joint Ethics Committee. HDBR is regulated by the UK Human Tissue Authority (HTA; www.hta.gov.uk) and operates in accordance with the relevant HTA Codes of Practice.

Zebrafish work was conducted in compliance with animal ethics committees at the Peter MacCallum Cancer Centre and University of Melbourne. All mouse procedures were approved by the German Federal Authorities (LaGeSo, Berlin; license ZH120) and conducted in accordance with institutional, state, and government regulations.

### Data availability.

Source data are provided in the Supporting Data Values file. Data used to generate Figure 3B (23), Supplemental Figure 2 (25, 26), and Supplemental Figure 3, D–G (27), were derived from sources in the public domain.

## Author contributions

SJ, PM, SM, SMA, and PO were responsible for conceptualization. SMA and PO were responsible for project administration. KO, RYB, IMC, RCSB, MM, SU, NRH, ES, AA, CK, KG, TM, and SMA conducted the investigation. KO, SED, ES, DG, JCDRJ, KSO, BMH, TM, RH, SMA, and PO performed the formal analysis. MO, DW, AE, KK, KG, BMH, RH, SM, and PO provided resources. KO, SMA, and PO wrote the original draft. All authors reviewed and edited the manuscript. SJ, PM, KSO, BMH, TM, RH, SM, SMA, and PO supervised the study. SJ, PM, SM, and PO acquired funding.

## Funding support

- British Heart Foundation (BHF; SP/13/5/30288 and FS/15/39/31526).
- A joint grant from the MRC and the BHF (MR/P011543/1 and RG/17/7/33217).
- A research fellowship from the Rosetrees Trust (St Georges-21\2) and St George’s Hospital Charity (RES 20-21 003) to SMA.

## Supplementary Material

Supplemental data

Unedited blot and gel images

Supplemental video 1

Supplemental video 2

Supporting data values

## Figures and Tables

**Table 1:**
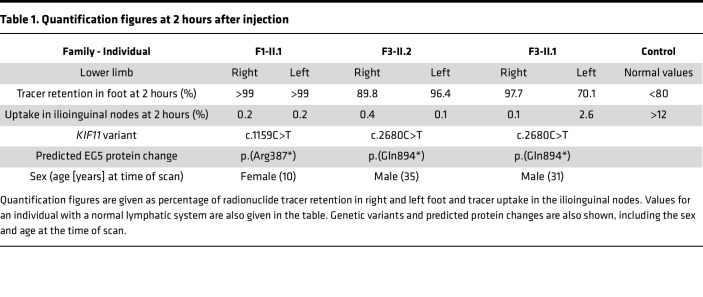
Quantification figures at 2 hours after injection

## Version 1 12/22/2025

In-Press Preview

## Version 2 02/09/2026

Electronic publication
