# Supplementary material for: Insights into KIF11 pathogenesis in microcephaly-lymphedema-chorioretinopathy syndrome from a lymphatic perspective
Source: JCI Insight. 2025 Dec 18;11(3):e177656. doi: 10.1172/jci.insight.177656 (PMC12893108; doi:10.1172/jci.insight.177656)
Supplement: Supplemental data [file jciinsight-11-177656-s216.pdf]

Insights into *KIF11* pathogenesis in Microcephaly-Lymphedema-Chorioretinopathy syndrome from a lymphatic perspective.

Kazim Ogmen[1], Sara E. Dobbins[1], Rose Yinghan Behncke[2,3], Ines Martinez-Corral[4#], Ryan C.S. Brown[5], Michelle Meier[5, 6, 7], Sascha Ulferts[2], Nils Rouven Hansmeier[2,8], Ege Sackey[1,9], Ahlam Alqahtani[10], Christina Karapouliou[1§], Dionysios Grigoriadis[1], Juan C. Del Rey Jimenez[9], Michael Oberlin[11], Denise Williams[12], Arzu Ekici[13], Kadri Karaer[14], Steve Jeffery[1], Peter Mortimer[1,15], Kristiana Gordon[1,15], Kazuhide S. Okuda[5,6‡], Benjamin M. Hogan[5,6], Taija Mӓkinen[4,16,17], René Hägerling[2,3,8], Sahar Mansour[1,9*], Silvia Martin-Almedina[1*], Pia Ostergaard[1]

Supplemental Methods

Lymphoscintigraphy

Three patients underwent lower limb lymphoscintigraphy, which is the imaging of the lymphatic system. This was performed according to standard local procedure by injecting a radioactive isotope (technetium-99m-nanocoll) into the web spaces between the toes (1) and imaging the uptake into the inguinal lymph nodes after 2h with a gamma camera. Quantification figures 2h post-injection were calculated as percentage (%) of tracer retention within the feet, and tracer uptake in the ilioinguinal nodes. Lymphoscintigraphy images have been provided by Dr Sue Heenan (St George's University Hospitals NHS Foundation Trust, London, UK). These were compared against a historical standard taken from authors' archive: "Methods of imaging the lymphatic system" by City St. George's University of London licensed under CC BY-SA-4.0.

Mouse embryo collection, mouse tissue collection, histology and immunofluorescence

Mice used in this study have been sourced from the animal facility of the MPI for Molecular Genetics and work was approved by German Federal Authorities (LaGeSo Berlin) under the licence number ZH120.

Wild-type mouse embryos (C57BL/6) were analyzed at developmental stages E10.5 to E13.5, with the embryonic day (E) determined by the appearance of a vaginal plug (E0.5), and ears and small intestines from eight-week-old wild-type mice (C57BL/6) were examined. Wild-type embryos collected between E10.5 and E13.5 were fixed in 4% paraformaldehyde (PFA) in PBS at room temperature for 2–4 hours (depending on sample size), washed twice in PBS, and then incubated sequentially in increasing sucrose solutions (5%, 10%, and 15% [w/v] in PBS) for one hour each (or until the embryos sank). Next, embryos were incubated in a 1:1 solution of 15% sucrose/PBS and Tissue-Tek® O.C.T. Compound (Sakura, 94-4583) for one hour at room temperature. Fully embedded embryos were snap-frozen in a chilled ethanol bath and stored at –80°C until sectioning. For standard histology, 5 µm

cryosections were fixed in 4% PFA/PBS for 15 minutes, washed in PBS, stained with hematoxylin and eosin for 1 minute, and then mounted with Entellan (Sigma-Aldrich, 1.07960); images were captured using a Zeiss Axio Observer Z7 epi-fluorescence microscope (20× air objective, NA = 0.8). For immunofluorescence, 10 μm cryosections were fixed in ice-cold methanol for 15 minutes, washed in PBS, and blocked for 1 hour at room temperature in a solution containing 10% chicken serum and 0.3% Triton X-100 in PBS; sections were then incubated for 1 hour with primary antibodies (diluted in 1% BSA, 1% chicken serum, and 0.3% Triton X-100 in PBS), washed three times in PBS-T (0.1% Tween-20 in PBS), and incubated with Alexa dye–conjugated secondary antibodies, followed by three additional washes in PBS-T, counterstaining with Hoechst, mounting in Fluorescence Mounting Medium (Dako Agilent, S3023), and imaging with a Zeiss LSM 980 confocal microscope (25× oil objective, NA = 0.8). For whole-mount immunofluorescence, 1 cm piece of mouse small intestine and ear were fixed in 4% PFA/PBS at room temperature for 4 hours, washed twice in PBS, permeabilized in 0.5% Triton X-100/PBS, and blocked in 1% BSA with 0.5% Tween-20/PBS—each step performed for 12 hours at 4°C; samples were then incubated with primary antibodies (diluted in 1% BSA and 0.5% Tween-20 in PBS) for 48 hours, washed three times in PBS-T, incubated with Alexa dye–conjugated secondary antibodies for another 48 hours, washed three times in PBS-T, mounted in Fluorescence Mounting Medium, and imaged using a Zeiss LSM 980 confocal microscope (25× oil objective, NA = 0.8). The antibodies used were Hoechst 33342 (62249, Waltham, MA, USA), rat monoclonal IgG2a anti-mouse PECAM-1 (102502, BioLegend, San Diego, CA, USA), rabbit polyclonal IgG anti-mouse PROX-1 (102-PA32AG, ReliaTech, Wolfenbüttel, DE), goat polyclonal IgG anti-mouse VEGFR3 (AF743, R&D Systems, Minneapolis, MN, USA), rabbit monoclonal IgG anti-rabbit Ki67 (GTX16667, GeneTex, Irvine, CA, USA), mouse monoclonal IgG anti-mouse KIF11 (627802, BioLegend, San Diego, CA, USA), donkey polyclonal anti-mouse IgG Alexa Fluor 488 (A21208, Invitrogen, Waltham, MA, USA), donkey polyclonal anti-rabbit IgG Alexa Fluor 568 (A10042, Invitrogen, Waltham, MA, USA), and donkey polyclonal anti-goat IgG Alexa Fluor 647 (A32849, Invitrogen, Waltham, MA, USA). This protocol ensured high-quality preservation and visualization of embryonic and adult lymphatic structures for subsequent analysis.

RNAscope in situ hybridization in mouse embryos

Mouse embryos were collected at different developmental stages between E10.5 and E14.5. They were fixed for 4 hours in 4% PFA/PBS at room temperature. The embryos were washed twice in PBS before incubation in an increasing sucrose series (5%, 10% and finally 15% (w/v) sucrose (Roth/PBS) each for an hour or until the embryos sank to the bottom of the tube. Finally, the embryos were incubated in 15% (w/v) sucrose/PBS and Tissue-Tek®

O.C.T. Compound (94-4583, Sakura) in a 1:1 solution before embedding the embryos in Tissue-Tek® O.C.T. Compound in a chilled ethanol bath and stored at -80°C for sectioning. The embryos were cut into 5 µm thick sections on slides for RNAscope. Simultaneous RNA *in situ* hybridization was performed using the RNAscope® technology (Advanced Cell Diagnostics [ACD]) and the following probes specific for Mm-Flt4 (Cat. No. 481371, ACD) and Mm-Kif11 (Cat. No. 819941-C3, ACD) on five µm sections of the mouse embryos. RNAscope probes were purchased and designed by ACD. The RNAscope® assay was run on a HybEZ™II Hybridization System (Cat. No. 321720, ACD) using the RNAscope® Multiplex Fluorescent Reagent Kit v2 (Cat. No. 323100, ACD) and the manufacturer's protocol for fixed-frozen tissue samples with target retrieval on a hotplate for 5 minutes. Fluorescent labelling of the RNAscope® probes was achieved by using OPAL 520, OPAL 570 and OPAL 690 dyes (Cat. No. FP1487001KT, Cat. No. FP1488001KT, Cat. No. FP14970014KT, Akoya Biosciences, Marlborough, MA, USA) and stained sections were scanned using an LSM 980 with Airyscan 2 at various magnifications (Carl Zeiss AG, Oberkochen, DE). For quantification of mRNA signal RNAscope probes specific for *Flt4* and *Kif11* were used. The number of signal-positive spots per area (25µm^2) for each probe was determined by manual counting. For each image up to 8 areas were analysed using ImageJ (2).

Zebrafish husbandry, genome editing, Ispinesib treatment, imaging and quantification
Zebrafish work was conducted in compliance with animal ethics committees at the Peter MacCallum Cancer Centre and The University of Melbourne. All fish lines used in this manuscript were sourced from The University of Melbourne. The following transgenic lines were used: *Tg(fli1a:nEGFP)^{y7}* (3) and *Tg(-5.2lyve1b:DsRed2)^{nz101}* (4). A guide RNA (gRNA) target was designed using CHOPCHOP (5) to target a highly conserved sequence within Exon 9 of the *kif11* gene and ordered from Integrated DNA Technologies (IDT). gRNA preparation was completed as previously described (6). gRNA: 5'-taatacgactcactata*GGTGAGAACATCGGACGATC*gttttagagctagaaatagc-3'. ~250ng/ul of the gRNA were co-injected with 0.5ug of Cas9 protein to AB. F0 adults were outcrossed and germ line transmission of the mutation was identified by polymerase chain reaction (PCR). Genotyping primers: 5'-GCTTTCGAGTCAGTGGGGTT-3', 5'-CCTGCAGTATGCGGGTTAGT-3'.

For inhibitor treatments, Tg(*fli1a:nEGFP;lyve1b:DsRed2*) embryos were incubated in E3 embryo medium containing 10, 25, 50, 75 or 100µM Ispinesib from either 10–48 hpf, to examine global phenotypes and survival, or 24–72 hpf, to examine endothelial cell defects. Drug treatments were performed in 6-well plates and kept at 28°C. All embryos were mounted in 0.5% low meting agarose (Merck, Darmstadt, Germany; A9414-100G) as

previously described (7). Imaging was completed at the Centre for Advanced Histology and Microscopy (Peter MacCallum Cancer Centre). Images in Figure 6A were taken on an Olympus BX53 microscope (Differential inference contrast (DIC) imaging, 10x Objective). The Nikon SoRa Spinning Disk Confocal was used for all remaining zebrafish imaging. The 10x objective was used for brightfield images Figure 6, B-C, and 20x objective used for fluorescent imaging in Figure 6D and Supplemental Figure 3B**.** Fluorescent images were stitched together to visualise the whole embryo. Endothelial cells (fli1a:nEGFP) were manually quantified with FIJI, Image J (National Institutes of Health) (8). Statistics were completed for these images as previously described (9).

ChIP-Seq data analysis

FOXC2, NFATC1 and PROX1 ChIP-Seq data was downloaded from NCBI sequence read archive (SRA) under accession number SRP191993, and GATA2 from the European Nucleotide Archive under accession number PRJEB9436. Data was processed using trim galore (https://www.bioinformatics.babraham.ac.uk/projects/trim_galore/), fastqc (http://www.bioinformatics.babraham.ac.uk/projects/fastqc) and aligned to human reference genome (b38) with Bowtie2 (v2.5.0) (10). Alignments were visualized using the Integrative Genomics Viewer (2.16) (11).

scRNA-seq data processing and analysis

Zebrafish: The previously published scRNA-seq data set (12) was processed as described in the original publication (9). Downstream analysis and visualisation were performed in R (v4.2.0) using built in functions in Seurat (v3.0). Pair-wise gene Spearman correlations were calculated with log normalised gene counts using the cor.test function from the stats package.

Mice: Two published scRNA-seq datasets of lymphatic endothelial cells (13, 14) were interrogated using webservers: (https://makinenlab.shinyapps.io/DermaLymphaticEndothelialCells/ and https://singlecell.broadinstitute.org/single_cell/study/SCP1821). *Kif11* was selected as the gene of interest and UMAP plots downloaded. Box plots of *Kif11* by cell type were generated and where possible, raw counts of total number of cells, and number of cells expressing *Kif11* extracted to perform a pairwise chi-sq test for association in R (v4.2.0).

Tyrosine Kinase (TK) array and Bioinformatic Analysis

The Human Phospho-Kinase Array (#ARY003B, R&D systems) was hybridised following the manufacturer's recommendations. Briefly, membranes containing captured antibodies against 43 kinases in duplicate, were incubated with either siRNA control or siRNA *KIF11* treated LECs lysates collected 24 hours post-transfection. Chemiluminescence was used for the

detection of antigen-antibody complexes, generating images for different exposure times. Signals were quantified using Image J (2) and results plotted with GraphPad Prism version 9.5.1 for Windows.

For the two duplicates the average Fold Change (FC) and the average Coefficient of Variation (CoV) were calculated for the normalised control and treatment levels for each kinase. Kinases were selected as dysregulated where FC>1.1 or FC<0.9, and where the average CoV was smaller compared to the observed variation between treatment and control, (plus FC effect must be twice as large as the average CoV for that kinase) at either the 1min or 10min chemiluminescence exposure timepoints. Selected kinases were excluded which were not consistent in the direction of change across all time points analysed. Interactions between the dysregulated kinases were identified using STRING (v11.5) (15) based on experimentally verified interactions. Cytoscape (v3.8.2) (16) was used to visualise the protein networks.

RNA isolation from LECs and RT-qPCR

RNA was isolated from LECs with the miniRNA extraction kit (Qiagen). 500 ng of total RNA was used as template for cDNA generation using Superscript III and Oligo dT (Invitrogen). Oligonucleotides are listed in Supplemental Table 4, and all efficiencies were confirmed above 95% in a standard curve analysis. Real-time PCR was performed using SYBR Green (Promega) and Bio-Rad CFX96 thermocycler. The results were analysed using Bio-Rad CFX Manager Software version 2.0. Quantification was done with the ΔΔCT method to calculate fold-differences with the reference gene *GAPDH*.

Immunofluorescence and Western blot

For immunofluorescence LECs grown on fibronectin-coated glass coverslips were fixed in 4% PFA for 15 minutes and permeabilized with 0.5% Triton X-100 for 5 minutes at room temperature. Blocking was done with 1% BSA for 1 hour before incubation with primary antibodies (EG5 1:50 ab72413, Abcam and α-tubulin (DM1A) 1:200 T9026, Sigma) overnight at 4ºC. Coverslips were then incubated with secondary antibodies (anti-rabbit Alexa Fluor 488 1:500 A-21206 and anti-mouse Alexa Fluor 555 1:500 A31570, Life Technologies) for 1 hour at room temperature, DAPI (Cat # 422801, Biolegend) for 5 minutes and mounted in Vectashield (Vector Laboratories). Images were taken with a Zeiss Axiovert 200M in the City St George's University of London Image Research Facility.

For western blot LECs or lymphoblastoid cell lines were harvested in lysis buffer (20 mM Tris pH 7.5, 150 mM NaCl, 0.5%Triton X-100) supplemented with protease and phosphatase inhibitors (Sigma-Aldrich). After clarification by centrifugation, protein lysates were separated by SDS-PAGE and transferred to PVDF-P membranes that were blocked with 5% milk or BSA in PBS. Immunoblot analysis was performed with the following primary antibodies: anti-human

EG5 (1:1000, ab72413-Abcam, CC10014-Cell Applications, NB500-181-Novus Biologicals), anti-human GAPDH (1:10000, MAB374, Merck Millipore), anti-human PROX1 (1:1000, AF2727, Biotechne), anti-human VEGFR3 (1:50, MAB3757, Merck Millipore), anti phospho-Akt (Ser473) (1:1000, #4051, Cell Signalling), anti Akt (1:1000, #9272, Cell Signalling), anti-phospho-p44/42 MARPK (Erk1/2) (Thr202/Tyr204) (1:1000, #9106, Cell Signalling), anti p44/42 MARPK (Erk1/2) (1:1000, #9102, Cell Signalling). Secondary antibodies were HRP-conjugated and chemiluminescence detection was performed with ECL™ (GE Healthcare). Protein expression was quantified by densitometry and normalized against GAPDH using Image J.

Transwell migration assay

LECs were serum starved for 16 hours prior to seeding into 8 μm cell culture transwell inserts (BD Biosciences) placed in 24-well plates (Santa Cruz Biotechnology, USA) and coated with fibronectin. Prior to seeding cells, inserts were filled with PBS (with Ca^{2+} and Mg^{2+}) and incubated for an hour. $5x10^4$ cells per insert were plated in endothelial medium MV2 and the same medium supplemented with 100ng/mL VEGFC was added to the lower chamber. After 10 hours, inserted membranes were fixed in methanol at -20°C and nuclei stained with DAPI. After removing residual cells in the upper chamber with a cotton swab, nuclei on the lower surface of the membrane were counted using an EVOS M5000 digital inverted microscope (10X). An average count of 5 different fields of view were quantified.

Wound healing assay

LECs were seeded within 2-well culture inserts (Cat # 80209, ibidi, Germany) attached to fibronectin-coated glass bottomed plates at a density of 34,000 cells/insert chamber. After 2 hours, the insert was removed, and cells were washed twice with PBS containing Ca^{2+} and Mg^{2+} before exposure to either vehicle (DMSO 0.01%) or 50nM Ispinesib in endothelial cell growth medium MV2 with 100ng/mL VEGFC.

Single cell migration tracking

Quantitative phase image microscopy assays were performed using a Livecyte microscope (Phasefocus Limited, UK) according to the manufacturer's instructions. In brief, LECs were plated and treated as described for wound healing assay (two independent experiments [#1 and #2] were performed using two distinct LEC single-donor batches). Images of wound closure were acquired every 10 minutes for 15 hours using a 10x objective lens at 37°C and 5% CO_2. Analysis of tracks to evaluate cell motility and speed was implemented using CellTrackR (v1.1.0) (17). Analysis of tracks was performed over time frames ($50 \geq t \leq 100$). Star plots were generated with the CellTrackR normalizeTracks feature selecting data with a minimum track length of 40. Thirty tracks were randomly selected to be displayed from the full

set using the R runif function. To reduce potential bias (18) velocity was calculated for each step for all cells and tracks. For experiment #1 the analysis was based on 2,270 control and 1,991 treatment tracks (44,686 and 43,613 steps). For experiment #2 the analysis was based on 6,676 control and 9,927 treatment tracks (89,804 and 97,713 steps). The difference between the distributions of speeds was assessed using the Wilcoxon-rank-sum test implemented in R. As an alternative metric for investigating cell motility, we also assessed the number of cells entering the scratch-window and the area of cells / scratch-window area across timeframes ($0>t<200$). The scratch window areas were defined by x = (300-500) for experiment #1 and x = (400-600) for experiment #2. One rogue cell with area >7,000μm² was removed from the analysis.

Spheroid sprouting assay

To generate cell spheroids (optimally ~750 cells per spheroid) LECs were trypsinized and suspended in growth factor supplemented MV2 medium without VEGFC and seeded in ultra-low adhesive round-bottomed 96-well plates (Cat # 4515, Greiner, Frickenhausen, Germany). After 24 hours at 37°C (5% CO_2), spheroids were harvested. Collagen stock solution was prepared prior to use by mixing 8 volumes (2mg/ml, 4°C) of rat tail collagen type-1 (Santa Cruz Biotechnology, USA) with 1 volume of 10X HBSS (Gibco, Thermo Fisher Scientific), and 1 volume of 0.2 N NaOH. Neutralised collagen solution was mixed with harvested spheroids (10-15 spheroids/condition). 150ng/mL VEGFC and 50nM Ispinesib were also added into a neutralised collagen and spheroids mix, before transferring to prewarmed tissue culture plates. After 30 minutes at 37°C to allow gel polymerization, 0.2mL MV2 medium containing growth factors was pipetted on top of each gel. After 6 hours representative images were taken and after 24 hours, in vitro sprouting was quantitated digitally (EVOSM5000) by measuring the number of the sprouts per spheroid and the length of each sprout (calculated and presented as average sprout length per spheroid).

Proliferation assay

LECs were transfected with siRNA Control or siRNA *KIF11* and after 24 hours proliferating cells were labelled with the Click-iT™ Edu Cell Proliferation Kit for Imaging, Alexa Fluor™ 647 dye (C10340, Thermo Fisher Scientific) following the manufacturer's instructions. Images were taken at 20x magnification using the Nikon A1R inverted confocal microscope (City St George's University of London Image Research Facility). For the quantification of LEC proliferation, one picture from the center of each sample was captured and the total number of nuclei, as well as the number of EdU positive cells, were counted using the Nikon NIS-Elements C software.

Supplemental Figures, Video and Tables

List of Supplemental Figures, Video and Tables

Supplemental Figure 1: Pedigrees of MLC families.

Supplemental Figure 2: Analysis of single cell transcriptomic data to interrogate *Kif11* expression.

Supplemental Figure 3: Additional analysis of zebrafish *kif11* function and expression.

Supplemental Figure 4: *KIF11* loss-of-function in in vitro models in human dermal lymphatic endothelial cells.

Supplemental Video 1: In vitro wound healing process of Lymphatic Endothelial Cells (LECs) captured using a label-free high content imaging system.

Supplemental Table 1: Clinical summary of MLC patients with confirmed *KIF11* variants.

Supplemental Table 2. Detailed clinical report.

Supplemental Table 3: Annotation and variant interpretation of the four *KIF11* variants identified in this study.

Supplemental Table 4: Oligonucleotides (5'- 3') used for RT-qPCR in Figure 8D and Supplemental Fig 4C.

Supplemental Figures

Figure S1

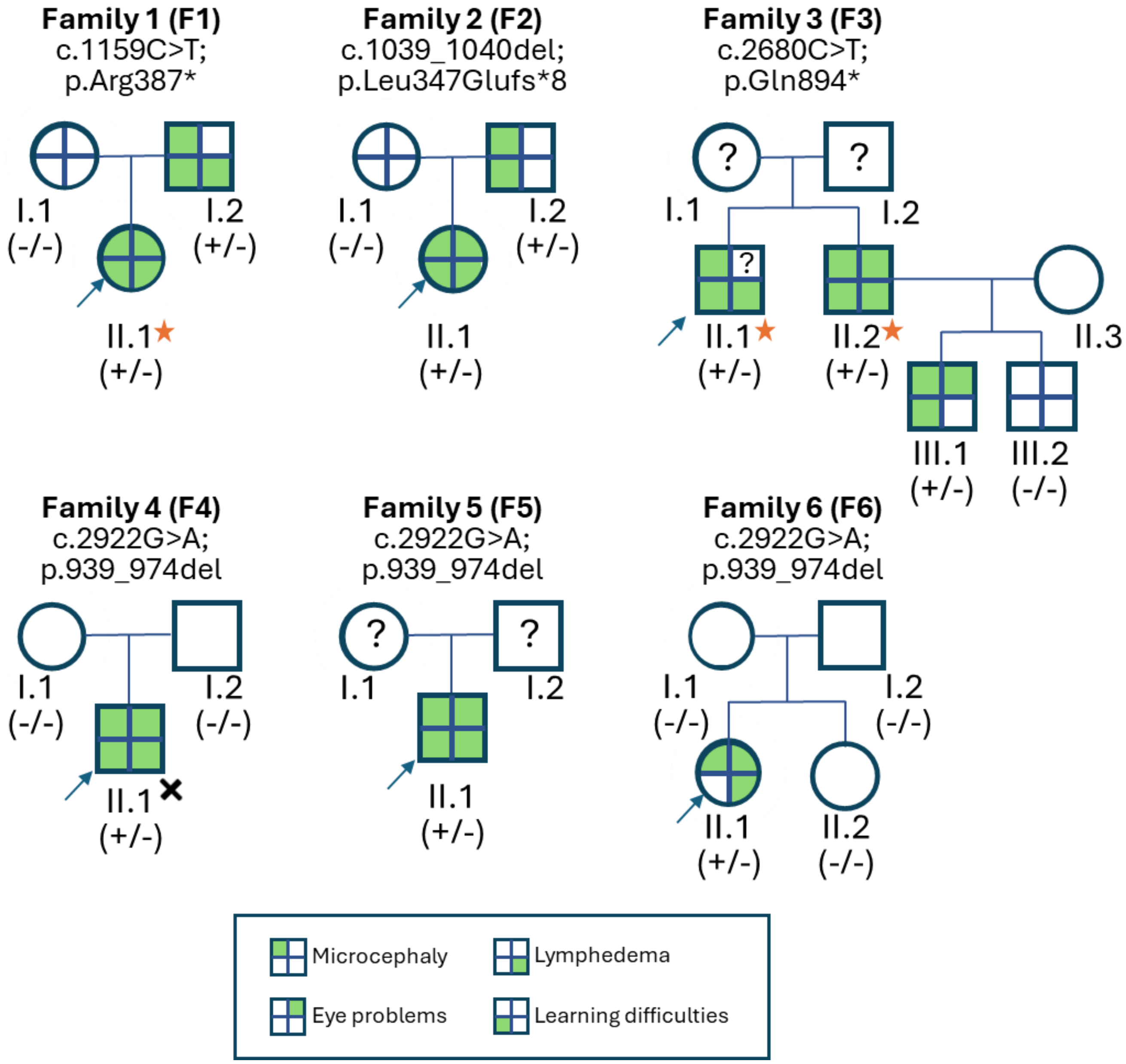

Supplemental Figure 1: Pedigrees of MLC families. The pedigrees of six families featuring *KIF11* variants are shown. Affected individuals are indicated with filled circles or squares. The probands are marked with arrows. *KIF11* genotypes are indicated for individuals who underwent exome or Sanger sequencing. The wildtype allele of the genotype is indicated by minus sign (-) and a plus (+) represents the alternative allele. The stars indicate the individuals who had a lymphoscintigraphy scan. The question marks represent individuals with unknown disease or condition status. The cross indicates the individual who underwent biopsy.

Figure S2

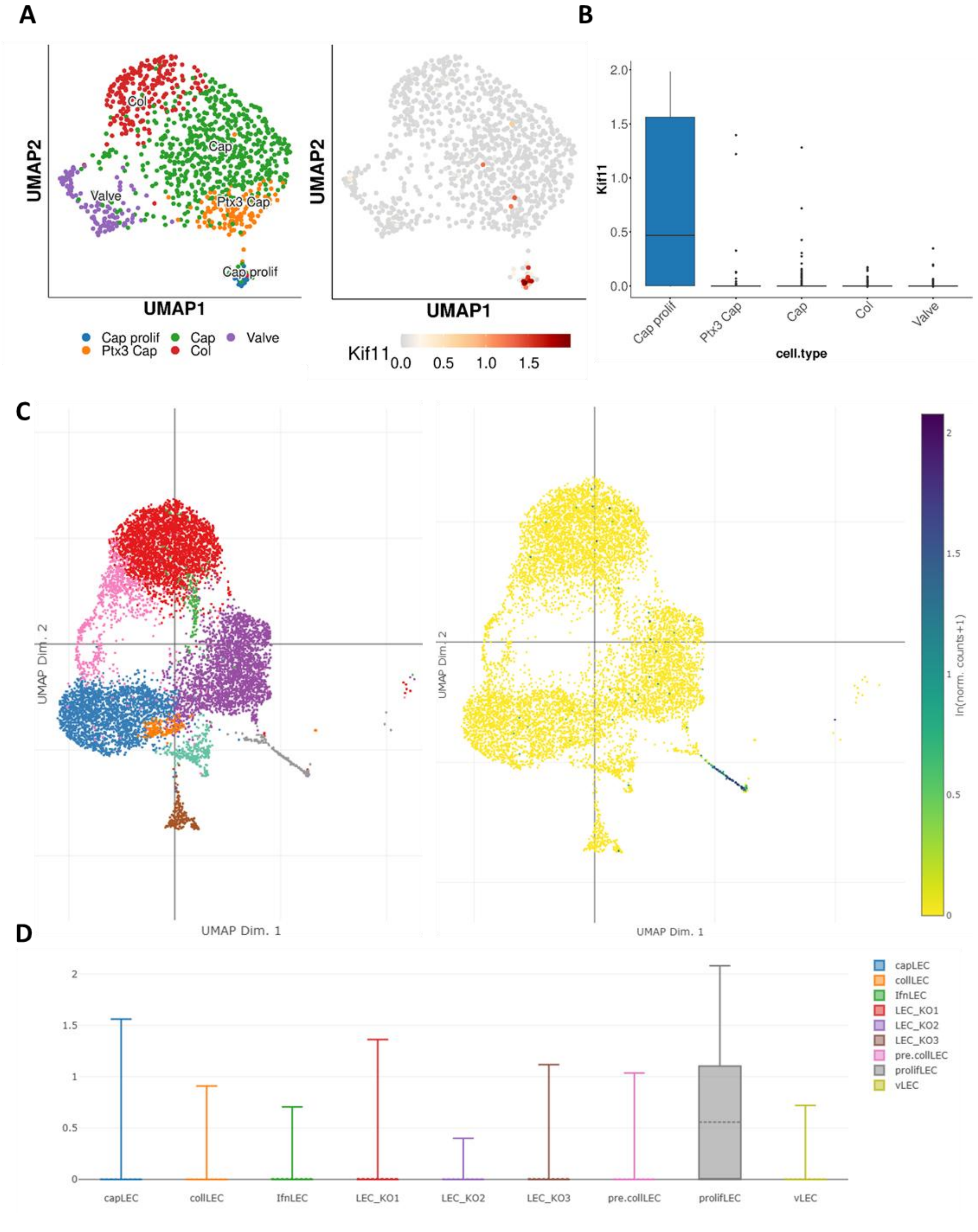
A
B
C
D
UMAP2
UMAP1
Col
Cap
Valve
Ptx3 Cap
Cap prolif
Cap prolif
Cap
Valve
Ptx3 Cap
Col
Kif11
0.0
0.5
1.0
1.5
2.0
Kif11
Cap prolif
Ptx3 Cap
Cap
Col
Valve
cell.type
UMAP Dim. 1
UMAP Dim. 2
ln(norm. counts+1)
capLEC
collLEC
IfnLEC
LEC_KO1
LEC_KO2
LEC_KO3
pre.collLEC
prolifLEC
vLEC

Supplemental Figure 2: Analysis of single cell transcriptomic data to interrogate *Kif11* expression. (A-B) Data based on single cell sequencing from control mice at 4/5wks (majority of proliferative cluster from 4 wk old mice) demonstrates *Kif11* expression is concentrated in proliferative LECs, with significantly increased proportion of cells expressing *Kif11* compared to other groups (pairwise chi-sq $P < 0.001$). Data taken from (14). **(C-D)** Data based on single cell sequencing of adult mesenteric LECs from 3WT and 3 *Foxc2*lecKO adult mice, showing proliferative LECs increased in proportion in *Foxc2*lecKO vs WT LECs. Data taken from (13). In D, y-axis represents "ln(normalized counts + 1)". Figures reproduced from:
(A-B): https://makinenlab.shinyapps.io/DermaLymphaticEndothelialCells/ and
(C-D): https://singlecell.broadinstitute.org/single_cell

Figure S3

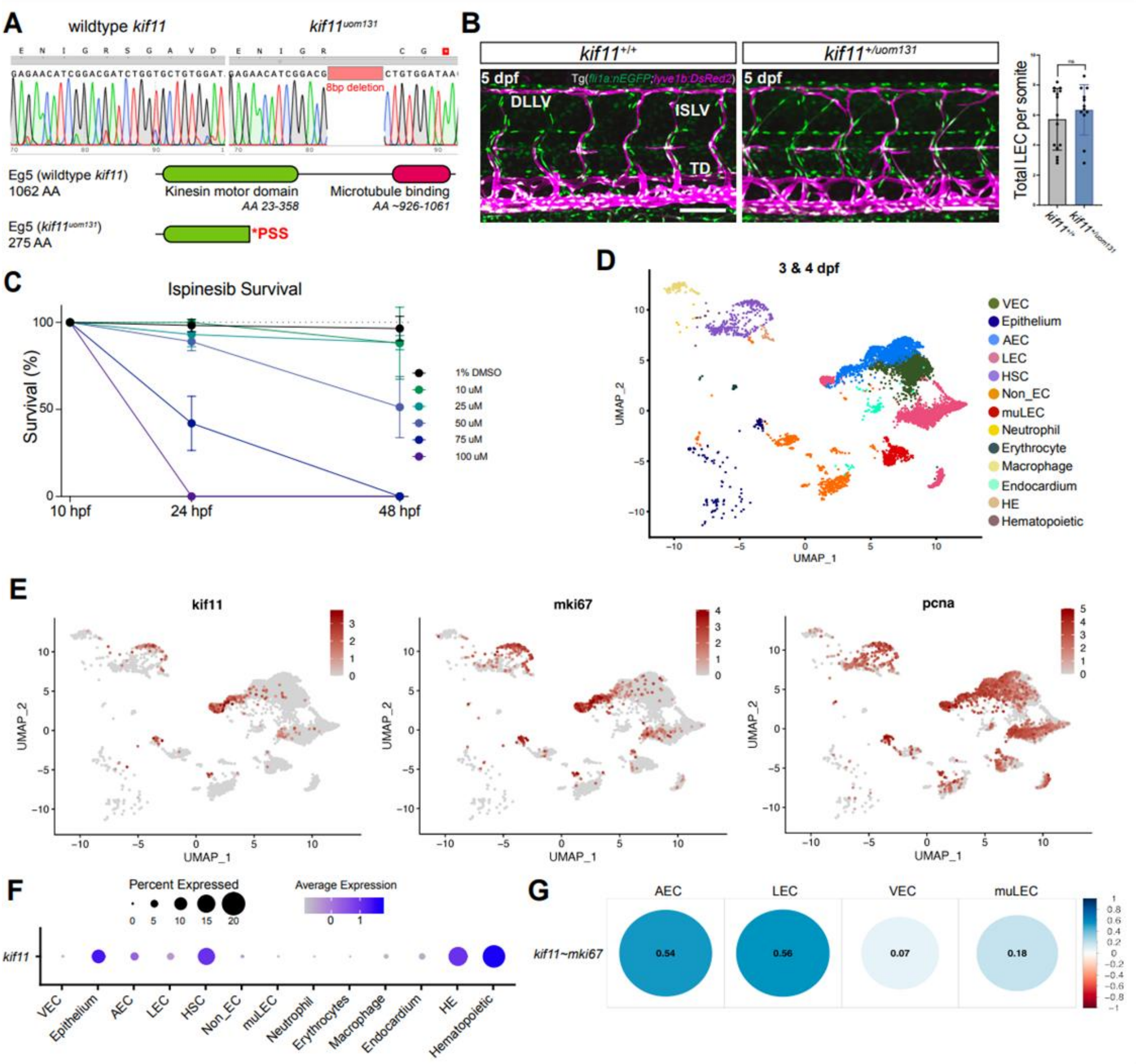
A
wildtype kif11
kif11uom131
8bp deletion
Eg5 (wildtype kif11)
1062 AA
Kinesin motor domain
AA 23-358
Microtubule binding
AA ~926-1061
Eg5 (kif11uom131)
275 AA
*PSS
B
kif11+/+
kif11+/uom131
5 dpf
Tg(fli1a:nEGFP;lyve1b:DsRed2)
DLLV
ISLV
TD
Total LEC per somite
ns
C
Ispinesib Survival
Survival (%)
10 hpf
24 hpf
48 hpf
1% DMSO
10 uM
25 uM
50 uM
75 uM
100 uM
D
3 & 4 dpf
UMAP_1
UMAP_2
VEC
Epithelium
AEC
LEC
HSC
Non_EC
muLEC
Neutrophil
Erythrocyte
Macrophage
Endocardium
HE
Hematopoietic
E
kif11
mki67
pcna
F
Percent Expressed
Average Expression
kif11
Erythrocytes
Hematopoietic
G
kif11~mki67
0.54
0.56
0.07
0.18

Supplemental Figure 3: Additional analysis of zebrafish *kif11* function and expression. **(A)** Upper: Chromatograms for wildtype and $kif11^{uom131}$ mutant zebrafish identifying an 8 bp deletion following CRISPR mutagenesis, predicted to induce a frameshift and subsequent premature stop codon. Lower: Predicted Eg5 protein structure for wildtype and $kif11^{uom131}$. The premature stop codon induces a truncation within the kinesin motor domain of Eg5 (https://www.ebi.ac.uk/interpro/protein/UniProt/F1QK82/). **(B)** Left: Lateral views of Tg(*fli1a:nEGFP;lyve1b:DsRed2)* 5 dpf wildtype and *kif11* heterozygous trunk vasculature with endothelial nuclei (green) and veins and lymphatics (magenta) labelled. Right: LEC numbers in *kif11* heterozygotes and controls (wildtype n=12, $kif11^{(+/uom131)}$ n=11). Homozygotes were unable to be analysed at this stage due to earlier lethality. Statistics: Mann-Whitney test. **(C)** Percentage survival of embryos treated with 1% DMSO, 10, 25, 50, 75 and 100μM Ispinesib-treated wildtype embryos from 10–48 hpf. 3 replicates of n=18-20 embryos per condition. **(D)** UMAP visualisation of scRNA-seq data from (12) displaying endothelial and related cell populations from wildtype zebrafish at 3- and 4-dpf. **(E)** UMAPs showing the expression of *kif11, mki67* and *pcna* across different cell types. **(F)** Dot plot displaying *kif11* expression across all reported cell types. The size of circles indicates the percentage of cells which express *kif11* and the shading, high to low expression. **(G)** Dot plot showing Spearman correlation of gene expression between *kiff11* and *mki67* within endothelial cell populations. Size of circle indicates significance value (all correlations p-value < 0.05) and blue to red shading indicates positive or negative correlations, respectively. Value inside dots gives level of correlation. Data in D-G sourced from (12). UMAP, uniform manifold approximation and projection; DLLV dorsal longitudinal lymphatic vessel; ISLV, intersegmental lymphatic vessel; TD, thoracic duct; VEC, venous endothelial cell; AEC, arterial endothelial cell; LEC, lymphatic endothelial cell; HSC, haematopoietic stem cell; muLEC, mural lymphatic endothelial cell; HE, hemogenic endothelium.

Figure S4

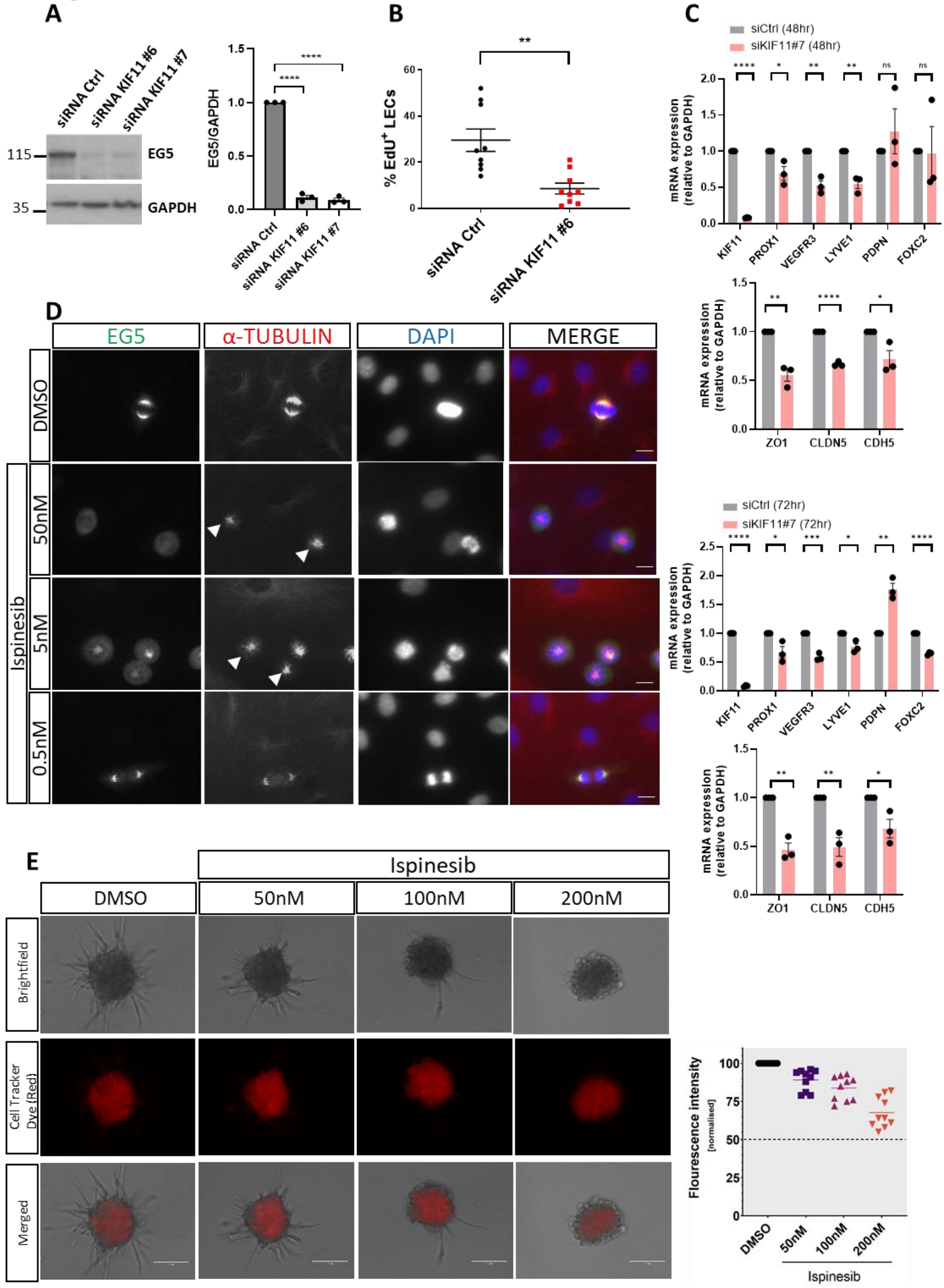
A
siRNA Ctrl
siRNA KIF11 #6
siRNA KIF11 #7
115
35
EG5
GAPDH
EG5/GAPDH
****
B
% EdU+ LECs
**
siRNA Ctrl
siRNA KIF11 #6
C
siCtrl (48hr)
siKIF11#7 (48hr)
siCtrl (72hr)
siKIF11#7 (72hr)
mRNA expression
(relative to GAPDH)
KIF11
PROX1
VEGFR3
LYVE1
PDPN
FOXC2
ZO1
CLDN5
CDH5
ns
D
EG5
α-TUBULIN
DAPI
MERGE
DMSO
50nM
5nM
0.5nM
Ispinesib
E
Ispinesib
DMSO
50nM
100nM
200nM
Brightfield
Cell Tracker
Dye (Red)
Merged
Flourescence intensity
[normalised]

Supplemental Figure 4: *KIF11* loss-of-function in in vitro models in human dermal lymphatic endothelial cells. (A) EG5 protein levels were assessed by western blot 24 hours post-transfection with siRNA *KIF11* or siRNA Ctrl (control). GAPDH used as loading control. One representative image is shown from n=3 experiments. Data represent mean ± SEM. ****P<0.0001. Two-tailed unpaired Student's t-test. **(B)** 24 hours after transfection with siRNA *KIF11* #6 or siRNA Ctrl, proliferating LECs were labelled with Alexa Fluor 647 after EdU incorporation and Hoechst 33342 for the staining of the total nuclei. Data represents mean ± SEM (n=3 biological replicates with 3 technical replicates each). **P<0.01. two-tailed unpaired Student's t-test. **(C)** *KIF11, PROX1*, *FLT4* (VEGFR3)*, LYVE1, PDPN, FOXC2*, *TJP1* (ZO1)*, CLDN5 and CDH5* gene expression relative to *GAPDH* in LECs treated with siRNA *KIF11* #7 for 48 and 72 hours analysed by qPCR. Bars represent mean relative expression ± SEM, n=3 experiments. ns=not significant, *P<0.05, **P<0.01, ***P<0.001. Two-tailed unpaired Student's t-test. **(D)** In DMSO (vehicle) treated cells, EG5 (green) and α-tubulin (red) co-localize in the bipolar spindle poles. Co-localization is lost in LECs treated with Ispinesib 50nM and 5nM (but not 0.5nM) and show abnormal monopolar spindle poles (arrow heads). Scale bar 10 µm, n=1. **(E)** CellTracker™ Red CMTPX staining of live 3D spheroids. The spheroids were stained for 2 hours with non-toxic ThermoFisher CellTracker at the end of the 3D spheroid assay where spheroids had been exposed to DMSO control or various concentrations of Ispinesib (50,100,200nM) for 24 hours. Spheroid images were captured by EVOS M5000 and fluorescence intensity of dye was measured (using ImageJ). Fluorescence dye directly indicates viable cells as the dye can only be retained in living cells which then can transfer to daughter cells but not adjacent cells. Representative images are shown from n=2 biological experiments from a total of 10 spheroids.

Supplemental Video

Supplemental Video 1: In vitro wound healing process of Human Dermal Lymphatic Endothelial Cells (HDLECs) captured using a label-free high content imaging system. Phase contrast video (black=background; white=LECs) of in vitro wound healing process of human primary lymphatic endothelial cells (LECs) being exposed to (a) 0.01% DMSO control or (b) 50nM ispinesib. MP4 video was created by capturing the wound healing process at 10-minute intervals for 15 hours. (25.00 frames/sec; frame width=768, height=768). Representative videos from n=2 biological experiments/per condition.

Supplemental Tables

Supplemental Table 1. Clinical summary of MLC patients with confirmed *KIF11* variants. For a more detailed clinical description go to Supplemental Table 2.

Family	ID	Sex	Age at last assessment (years)	Nucleotide variant	Bilateral lower limb lymphedema	Age of onset	Systemic involvement	Head circumference (SD)	Retinal changes or other eye features	Dysmorphic	Learning difficulties
F1	II.1	F	23	c.1159C>T	Y	Birth	N	-8.0	Y	Y	Mild
F1	I.2	M	56	c.1159C>T	Minimal		N	-2.0	N	N	Mild
F2	II.1	F	17	c.1039_1040del	Y	Birth	N	-5.7	Y	Y	Mild
F2	I.2	M	47	c.1039_1040del	N		N	-2.0	N	Mild	Mild
F3	II.1	M	31	c.2680C>T	Y	Birth	N	-3.2	(Y)	N	Moderate
F3	II.2	M	35	c.2680C>T	Y	Birth	N	-3.2	Y	N	Autism
F3	III.1	M	8	c.2680C>T	(N)			na	Y		Y
F4	II.1	M	7	c.2922G>A	Y	Birth	Y	-4.5	Y	Y	Mild
F5	II.1	M	28	c.2922G>A	Y	(Birth)		na	Y		Mild
F6	II.1	F	14	c.2922G>A	Y	<3 mth	N	-4.8	Y	Y	N

F, female; M, male; mth, month; N, no; na, microcephaly confirmed but details not available; SD, standard deviation; Y, yes; ( ), unconfirmed clinical finding.

Supplemental Table 2. Detailed clinical report. See Figure S1 for pedigrees of all MLC families.

**Family 1**	This case was first published as MLCRD01 in (19). The proband (F1-II.1) presented with congenital bilateral pedal oedema with large calibre veins. She was noted to be microcephalic antenatally and this was confirmed at birth. At the age of 4 years and 2 months, she had mild developmental delay and was dysmorphic with large, prominent ears, full cheeks and a broad nasal bridge. Her head circumference was 41.5cm (-8 SD) with a weight of 12.5kg (1st Centile, -2.4SD) and height of 94cm (2nd centile, -2.1 SD). Brain MRI was normal. No eye abnormalities were noted until the age of 12 years, when she was noted to have mild peripheral retinal pigmentation. At the age of 12 years, she also developed generalised myoclonic seizures and absences with an abnormal EEG. These were well controlled on lamotrigine. No systemic involvement was noted and there was no overgrowth. Lymphoscintigraphy at the age of 10 years showed bilateral functional aplasia with no lymphatic drainage demonstrated in either leg, either via the deep or the dermal lymphatics (Figure 1B).<br><br>Her father (F1-I.2) was noted to have mild bilateral lower limb lymphedema with prominent long saphenous veins and borderline microcephaly (just below the 3rd centile). He had been diagnosed with hypertrophic cardiomyopathy. He had no systemic involvement. The father has a squint and amblyopia but no retinal changes. His lymphoscintigraphy demonstrated a slightly sluggish uptake but was fundamentally normal. He was confirmed as carrying the pathogenic variant.
**Family 2**	This case was first published as MLCRD03 in (19). The proband (F2-II.1) was assessed at the age of 2 years. There had been an antenatal diagnosis of pericardial effusion and right ventricular hypertrophy which resolved before birth. At birth she was noted to have bilateral pedal oedema, with prominent saphenous veins and microcephaly. An echocardiogram demonstrated a thickened pulmonary valve that also resolved. At the time of assessment her head circumference was below the 0.4^{th} centile (-5.7 SD). She had mild developmental delay. Ophthalmic examination demonstrated a significant degree of hypermetropia and astigmatism. There were extensive atrophic patches in both fundi consistent with a diagnosis of *KIF11*-related chorioretinopathy (20).<br><br>Her father (F2-I.2) was noted to have borderline microcephaly with a head circumference between the 0.4^{th} and 2nd centile but with no evidence of lymphedema. He also struggled at school and probably had mild learning difficulties. He was confirmed as carrying the pathogenic variant.
**Family 3**	The proband (F3-II.1) presented in clinic at the age of 31 years with his brother. They were the only children of non-consanguineous parents of Indian origin. The father was said to have 'thick feet'. Proband had bilateral leg swelling, more prominent on the right leg than the left (Figure 1A).

	Prominent microcephaly and learning disability. He had a ten-year history of swelling predominantly of the right leg but with some swelling on the left. A deep vein thrombosis had been excluded. The oedema extended to his knee, it was firm and fibrotic, and the overlying skin was dry and hyperkeratotic. He had moderate learning difficulties, poor speech and autism and was cared for by his brother. He was described as having eye problems (unconfirmed by specialist). On examination he was microcephalic with a head circumference of 51.5cm (<0.4th centile; -3.2SD) and height of 163 cm. Lymphoscintigraphy showed functional aplasia on the right and abnormal tortuous tracts with patchy superficial re-routing on the left (Figure 1D). Genetic testing confirmed a heterozygous variant in *KIF11*. Parental DNA was not available to confirm inheritance.<br><br>The proband's brother (F3-II.2) is 35-year-old with bilateral lower limb lymphedema since childhood. The swelling of the feet was present at birth. He reported two episodes of cellulitis for which he had to be admitted to hospital. He had a diagnosis of autistic spectrum disorder and dyslexia but was the main carer for his brother. He also has hypermetropia, amblyopia and mild retinal dystrophy. On examination he had bilateral lymphedema (Figure 1A) and was microcephalic with a head circumference of 51.5cm (<0.4th centile; -3.2SD) with a height of 163 cm. Lymphoscintigraphy showed significantly reduced function with re-routing on the right and functional aplasia on the left (Figure 1C). He was confirmed as carrying the pathogenic variant.<br><br>The brother has two children, one of which is an affected (F3-III.1) 8-year-old boy with microcephaly, learning difficulties and hypermetropic astigmatism, chorioretinal atrophic lesions, as well as nyctalopia. He was also confirmed as carrying the pathogenic variant.
**Family 4**	The proband (F4-II.1) was a 7-year-old male born to non-consanguineous European parents. He was noted to be microcephalic antenatally and this was confirmed at birth. There was no family history of note. He had bilateral lower limb lymphedema (onset at birth) with primary microcephaly (head circumference 32cm at birth; -4.5SD), dysmorphic face, developmental delay, myopia, and an intermittent horizontal nystagmus. He was investigated for failure to thrive in early childhood with dysphagia and intermittent diarrhoea and vomiting (several times daily, independent of food intake and selection). Endoscopic investigations demonstrated a distal esophagitis and reflux esophagitis was histologically confirmed. In addition, the endoscopic changes seen were consistent with lymphangiectasia in the duodenum. Histologically, dilated lymph vessels were detected consistent with intestinal lymphangiectasia (Figure 1, F-G). In addition, the A1AT-value in the stool was significantly increased, consistent with a Protein Losing Enteropathy (PLE). This explains (in part) the failure to thrive and the intermittent diarrhoea. Currently he has a permanent feeding tube and on a diet without fat. A heterozygous variant

	was confirmed in *KIF11*, which was confirmed *de novo* after screening of parental DNA by Sanger sequencing.
**Family 5**	For proband (F5-II.1), very little information is available about his birth and pregnancy history, but he was noted to have microcephaly (no measurement available) and lymphedema shortly after birth. At the time, he had an MRI brain scan which is reported to have shown normal ventricles but with evidence of cerebral atrophy. Ophthalmic examination shows a reticular retinal abnormality. His paediatric notes at 18 months indicated that his development was within age-appropriate limits, although the need to monitor balance and comprehension was documented. He attended mainstream nursery and then mainstream school with support. He now attends special school and has significant learning difficulties and severe attention deficit hyperactivity disorder. Genetic testing confirmed a heterozygous variant in *KIF11*. Parental DNA was not available to confirm inheritance.
**Family 6**	This case was first published by Ekici and colleagues without a genetic report (21). In brief, proband (F6-II.1) was admitted to hospital with the complaint of microcephaly at 3 months. She was severely microcephalic, head circumference 34.6 cm (<5th centile; -4.8SD). She presented with dysmorphic facial appearance with broad nose with rounded tip, long philtrum with thin upper lip and prominent ears broad. She had bilateral oedema of the dorsum of the feet. Her developmental milestones were appropriate for her age. Ophthalmic examination at nine months of age showed bilateral chorioretinal changes and retinal pigmentation.<br><br>Cytogenetic analysis of her peripheral blood indicated a normal 46, XX karyotype, but as her phenotype fitted that of MLC, her DNA was screened for variants in *KIF11*. Sanger sequencing confirmed a heterozygous variant in *KIF11*, which was confirmed as *de novo* after screening of parental DNA.

Supplemental Table 3: Annotation and variant interpretation of the four *KIF11* variants identified in this study.

Family ID	Genomic coordinates (GRCh38)	Zygosity	Nucleotide change	Predicted protein change	SpliceAI (22)	CADD Phred score (23)	**ACMG/ACGS classification** (24, 25)			Reported by
							**Evidence**	**Pathogenicity Points**	**Classification**	
F1	chr10:92621415C>T	Heterozygous	c.1159C>T	p.Arg387*	NA	35	PVS1, PM2	10	Pathogenic	Ostergaard et al., 2012 (19)
F2	chr10:92616741-92616742del	Heterozygous	c.1039_1040del	p.Leu347Glufs*8	NA	33	PVS1, PM2	10	Pathogenic	Ostergaard et al., 2012 (19)
F3	chr10:92648344C>T	Heterozygous	c.2680C>T	p.Gln894*	NA	37	PVS1, PM2	10	Pathogenic	This study
F4<br>F5<br>F6	chr10:92649986G>A	Heterozygous	c.2922G>A	p.939_974del	0.31	22.8	PM2, PS4_Mod, PM6, PVS1(RNA_Mod) PS1_Mod†, PP4‡)	11	Pathogenic	This study and Schlögel *et al.*, 2015 (26).

Genomic coordinates (GRCh38), nucleotide and predicted protein changes are summarised. The variants were not present in gnomAD v4.1. Variant nomenclature is based on the reference sequence corresponding to *KIF11* isoform NM_004523.4 (MANE transcript).

Evidence points ranges: VUS: 0-5 (10-90% posterior probability pathogenicity); Likely pathogenic: 6-9 (90-99% posterior probability); Pathogenic: ≥10 (>99% posterior probability). Points awarded per evidence weighting sup (supporting) = 1, mod (moderate) = 2, str (strong) = 4, vstr (very strong) = 8 (ACGS 2024 variant interpretation guidelines: https://www.acgs.uk.com/media/12533/uk-practice-guidelines-for-variant-classification-v12-2024.pdf)

† An alternative variant at the same position with the same proven effect has been reported (27).
‡ Following the guidance of (24), PP4 supportive was applied based on the immuno-histological analysis of an intestinal biopsy from patient F4-II.1 (p.939_974del) (Fig. 1F-G), which showed aberrant morphology and significantly reduced lymphatic vessel density ($p < 0.01$) compared to controls.

Supplemental Table 4: Oligonucleotides (5'- 3') used for RT-qPCR in Figure 8D and Supplemental Figure 4C.

KIF11	Forward	AGCAAGCTGCTTAACACAGTT
	Reverse	CCTTCTTACGATCCAGTTTGGAA
GAPDH	Forward	CAAGGTCATCCATGACAACTTTG
	Reverse	GGGCCATCCACAGTCTTCTG
VEGFR3	Forward	TGCACGAGGTACATGCCAAC
	Reverse	GCTGCTCAAAGTCTCTCACGAA
PROX1	Forward	TACGCACGTCAAGCCATCAA
	Reverse	CAGGAATCTCTCTGGAACCTCA
TJP1	Forward	CGGTCCTCTGAGCCTGTAAG
	Reverse	GGATCTACATGCGACGACAA
CLDN5	Forward	CTCTGCTGGTTCGCCAACAT
	Reverse	CACAGACGGGTCGTAAAACTC
CDH5	Forward	GATCAAGTCAAGCGTGAGTCG
	Reverse	AGCCTCTCAATGGCGAACAC
PDPN	Forward	GGGAAGGTACTCGCCCTAAAG
	Reverse	CACGGGTCATCTTCTCCCAC
FOXC2	Forward	GGGGACCTGAACCACCTC
	Reverse	AACATCTCCCGCAGTTG
LYVE1	Forward	GCTTCAGCCTGGTGTTGCTT
	Reverse	GCCGGCCAAACTTAGTCCCA

References

1. Sarica M, Gordon K, van Zanten M, Heenan SD, Mortimer PS, Irwin AG, et al. Lymphoscintigraphic Abnormalities Associated with Milroy Disease and Lymphedema-Distichiasis Syndrome. Lymphat Res Biol. 2019 -12;17(6):610–9.

2. Schneider CA, Rasband WS, Eliceiri KW. NIH Image to ImageJ: 25 years of image analysis. Nat Methods. 2012 -07;9(7):671–5.

3. Lawson ND, Weinstein BM. In vivo imaging of embryonic vascular development using transgenic zebrafish. Dev Biol. 2002 -08-15;248(2):307–18.

4. Okuda KS, Astin JW, Misa JP, Flores MV, Crosier KE, Crosier PS. lyve1 expression reveals novel lymphatic vessels and new mechanisms for lymphatic vessel development in zebrafish. Development. 2012 -07;139(13):2381–91.

5. Montague TG, Cruz JM, Gagnon JA, Church GM, Valen E. CHOPCHOP: a CRISPR/Cas9 and TALEN web tool for genome editing. Nucleic Acids Res. 2014 -07;42(Web Server issue):401.

6. Gagnon JA, Valen E, Thyme SB, Huang P, Akhmetova L, Pauli A, et al. Efficient mutagenesis by Cas9 protein-mediated oligonucleotide insertion and large-scale assessment of single-guide RNAs. PLoS One. 2014;9(5):e98186.

7. Okuda KS, Baek S, Hogan BM. Visualization and Tools for Analysis of Zebrafish Lymphatic Development. Methods Mol Biol. 2018;1846:55–70.

8. Schindelin J, Arganda-Carreras I, Frise E, Kaynig V, Longair M, Pietzsch T, et al. Fiji: an open-source platform for biological-image analysis. Nat Methods. 2012 -06-28;9(7):676–82.

9. Stuart T, Butler A, Hoffman P, Hafemeister C, Papalexi E, Mauck WM, et al. Comprehensive Integration of Single-Cell Data. Cell. 2019 -06-13;177(7):1888,1902.e21.

10. Langmead B, Salzberg SL. Fast gapped-read alignment with Bowtie 2. Nat Methods. 2012 -03-04;9(4):357–9.

11. Thorvaldsdóttir H, Robinson JT, Mesirov JP. Integrative Genomics Viewer (IGV): high-performance genomics data visualization and exploration. Brief Bioinform. 2013 -03;14(2):178–92.

12. Grimm L, Mason E, Yu H, Dudczig S, Panara V, Chen T, et al. Single-cell analysis of lymphatic endothelial cell fate specification and differentiation during zebrafish development. EMBO J. 2023 -03-13:e112590.

13. González-Loyola A, Bovay E, Kim J, Lozano TW, Sabine A, Renevey F, et al. FOXC2 controls adult lymphatic endothelial specialization, function, and gut lymphatic barrier preventing multiorgan failure. Science Advances. 2021 July 16;7(29):eabf4335.

14. Petkova M, Kraft M, Stritt S, Martinez-Corral I, Ortsäter H, Vanlandewijck M, et al. Immune-interacting lymphatic endothelial subtype at capillary terminals drives lymphatic malformation. Journal of Experimental Medicine. 2023 January 23;220(4):e20220741.

15. Szklarczyk D, Gable AL, Nastou KC, Lyon D, Kirsch R, Pyysalo S, et al. The STRING database in 2021: customizable protein-protein networks, and functional characterization of user-uploaded gene/measurement sets. Nucleic Acids Res. 2021 -01-08;49(D1):D605–12.

16. Shannon P, Markiel A, Ozier O, Baliga NS, Wang JT, Ramage D, et al. Cytoscape: a software environment for integrated models of biomolecular interaction networks. Genome Res. 2003 -11;13(11):2498–504.

17. Wortel IMN, Liu AY, Dannenberg K, Berry JC, Miller MJ, Textor J. CelltrackR: An R package for fast and flexible analysis of immune cell migration data. ImmunoInformatics. 2021 Oct;1-2:100003.

18. Beltman JB, Marée AFM, de Boer RJ. Analysing immune cell migration. Nat Rev Immunol. 2009 -11;9(11):789–98.

19. Ostergaard P, Simpson MA, Mendola A, Vasudevan P, Connell FC, van Impel A, et al. Mutations in KIF11 cause autosomal-dominant microcephaly variably associated with congenital lymphedema and chorioretinopathy. Am J Hum Genet. 2012 -02-10;90(2):356–62.

20. Liu Y, Moore AT. Congenital focal abnormalities of the retina and retinal pigment epithelium. Eye (Lond). 2020 -11;34(11):1973–88.

21. Ekici, A., Karaer, K., Yimenicioglu, S., Carman, K., Ekici,M. Microcephaly-Lymphedema-Chorioretinal Dysplasia Syndrome: Two Case Reports. International Journal of Clinical Pediatrics. 2018;6(3-4).

22. Jaganathan K, Kyriazopoulou Panagiotopoulou S, McRae JF, Darbandi SF, Knowles D, Li YI, et al. Predicting Splicing from Primary Sequence with Deep Learning. Cell. 2019 -01-24;176(3):535,548.e24.

23. Schubach M, Maass T, Nazaretyan L, Röner S, Kircher M. CADD v1.7: using protein language models, regulatory CNNs and other nucleotide-level scores to improve genome-wide variant predictions. Nucleic Acids Res. 2024 -01-05;52(D1):D1143–54.

24. Brnich SE, Abou Tayoun AN, Couch FJ, Cutting GR, Greenblatt MS, Heinen CD, et al. Recommendations for application of the functional evidence PS3/BS3 criterion using the ACMG/AMP sequence variant interpretation framework. Genome Medicine. 2019 December 31;12(1):3.

25. Richards S, Aziz N, Bale S, Bick D, Das S, Gastier-Foster J, et al. Standards and guidelines for the interpretation of sequence variants: a joint consensus recommendation of the American College of Medical Genetics and Genomics and the Association for Molecular Pathology. Genet Med. 2015 -05;17(5):405–24.

26. Schlögel MJ, Mendola A, Fastré E, Vasudevan P, Devriendt K, de Ravel TJL, et al. No evidence of locus heterogeneity in familial microcephaly with or without chorioretinopathy, lymphedema, or mental retardation syndrome. Orphanet J Rare Dis. 2015 -05-02;10:52.

27. Guo Z, Huo X, Wu D, Hao B, Liao S. A Novel Variant of the KIF11 Gene, c.2922G>T, Is Associated with Microcephaly by Affecting RNA Splicing. Dev Neurosci. 2022;44(2):113–20.
