# Supplementary material for: Insights into KIF11 pathogenesis in microcephaly-lymphedema-chorioretinopathy syndrome from a lymphatic perspective
Source: JCI Insight. 2025 Dec 18;11(3):e177656. doi: 10.1172/jci.insight.177656 (PMC12893108; doi:10.1172/jci.insight.177656)

Full unedited gel for Figure 2C

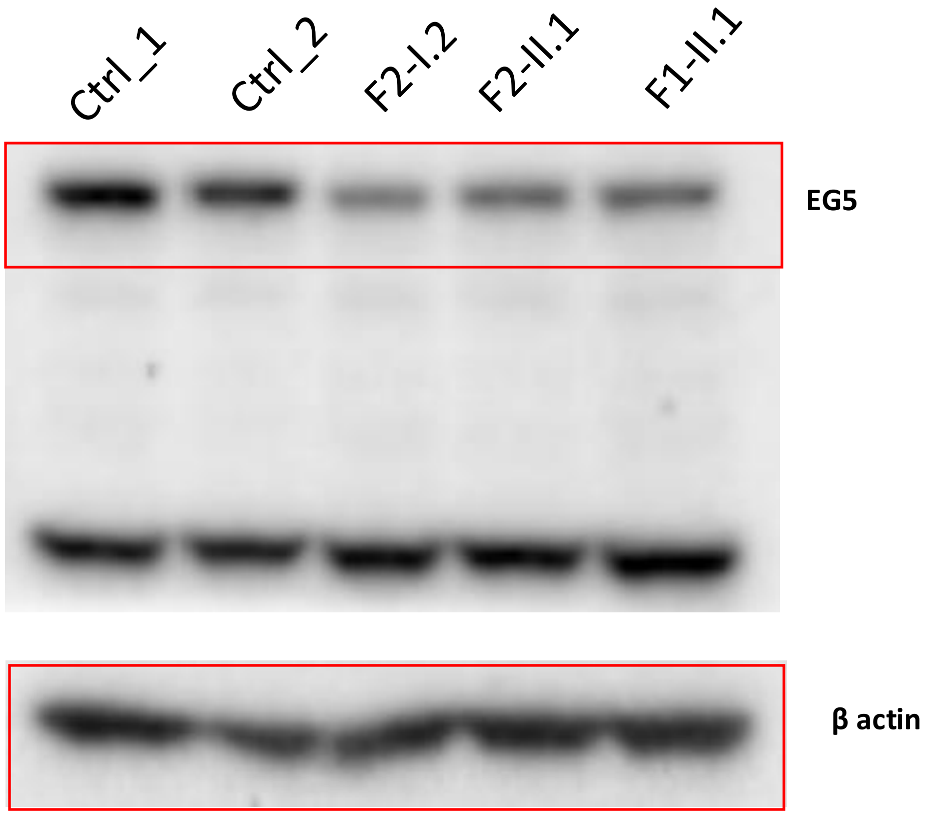

Full unedited gel for Figure 2D

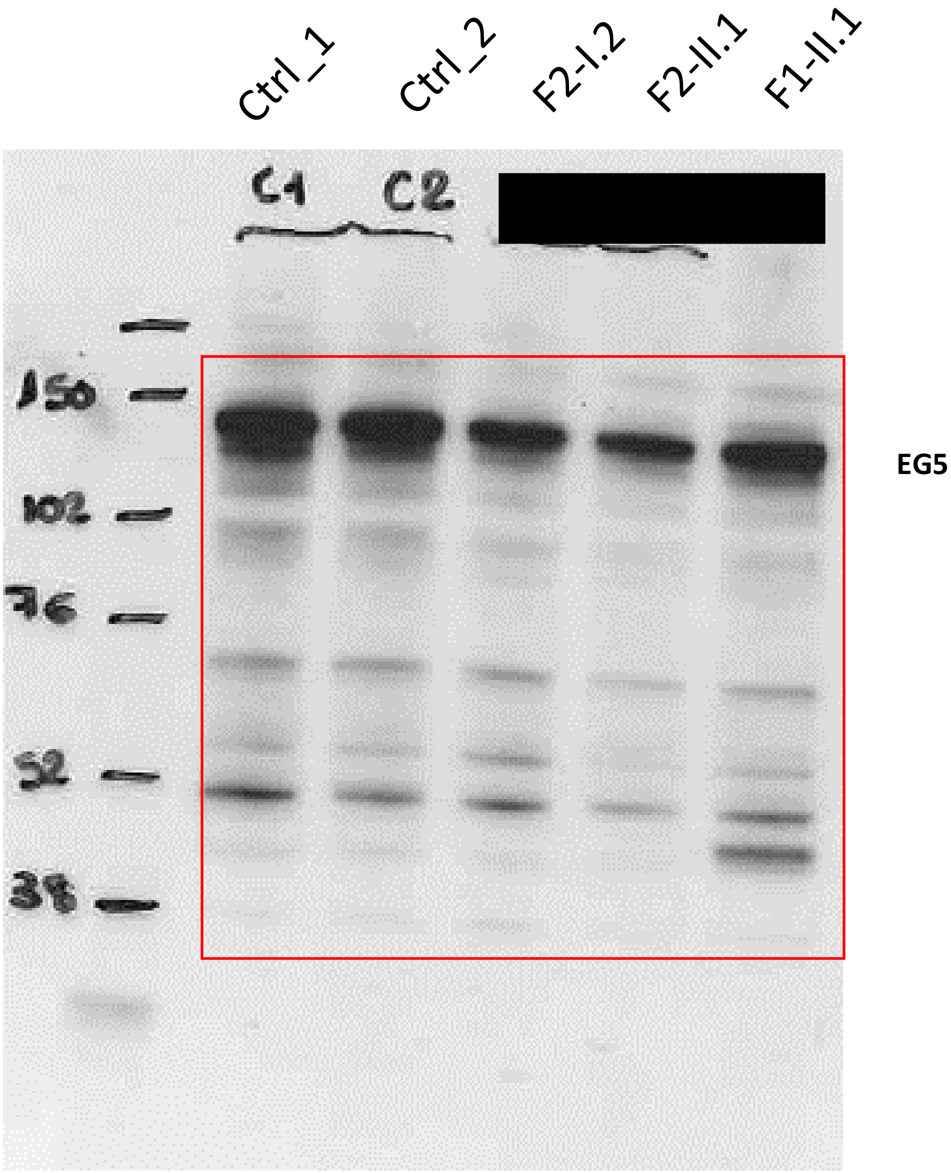

Full unedited gel for Figure 8A

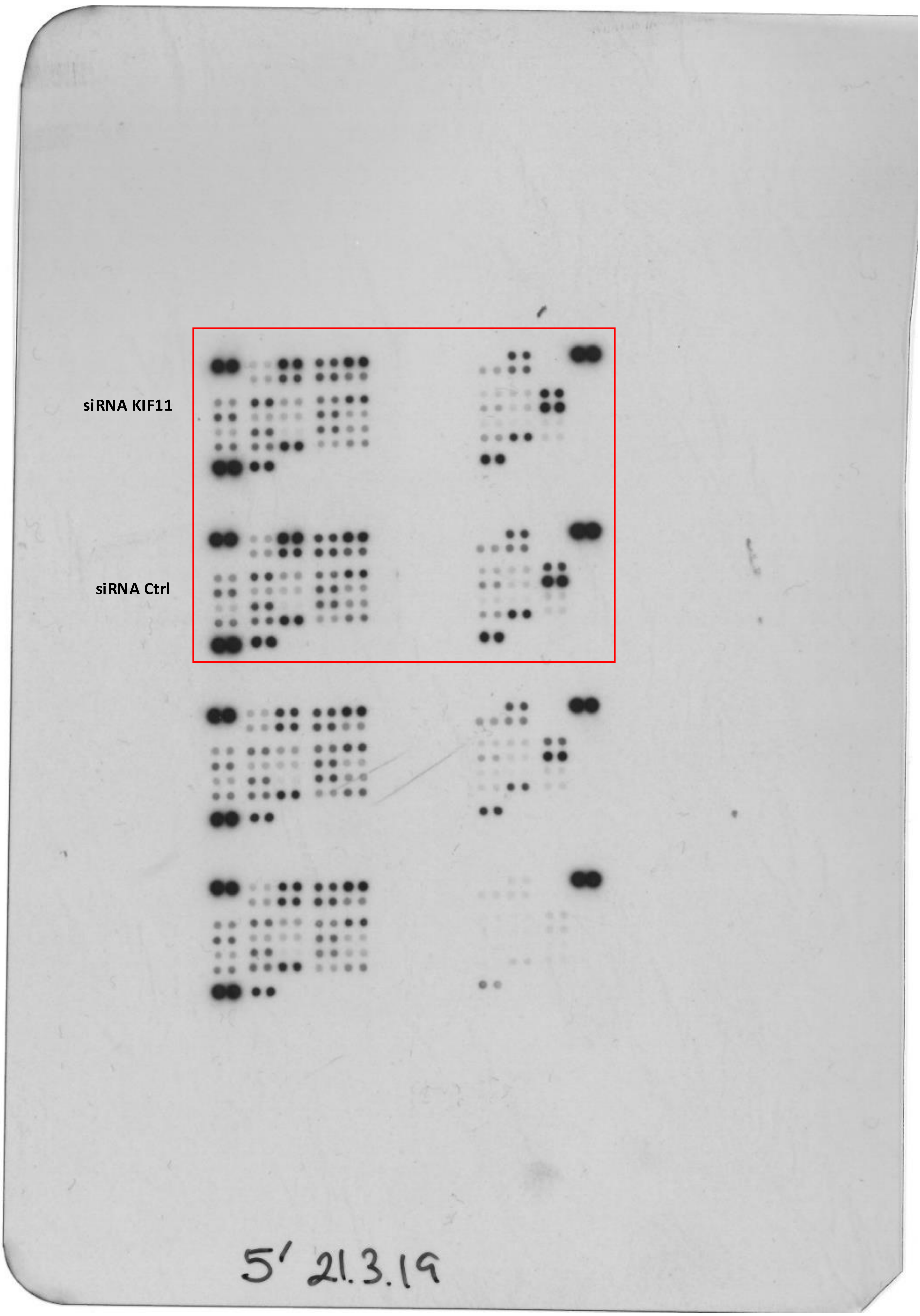

Full unedited gel for Figure 8C

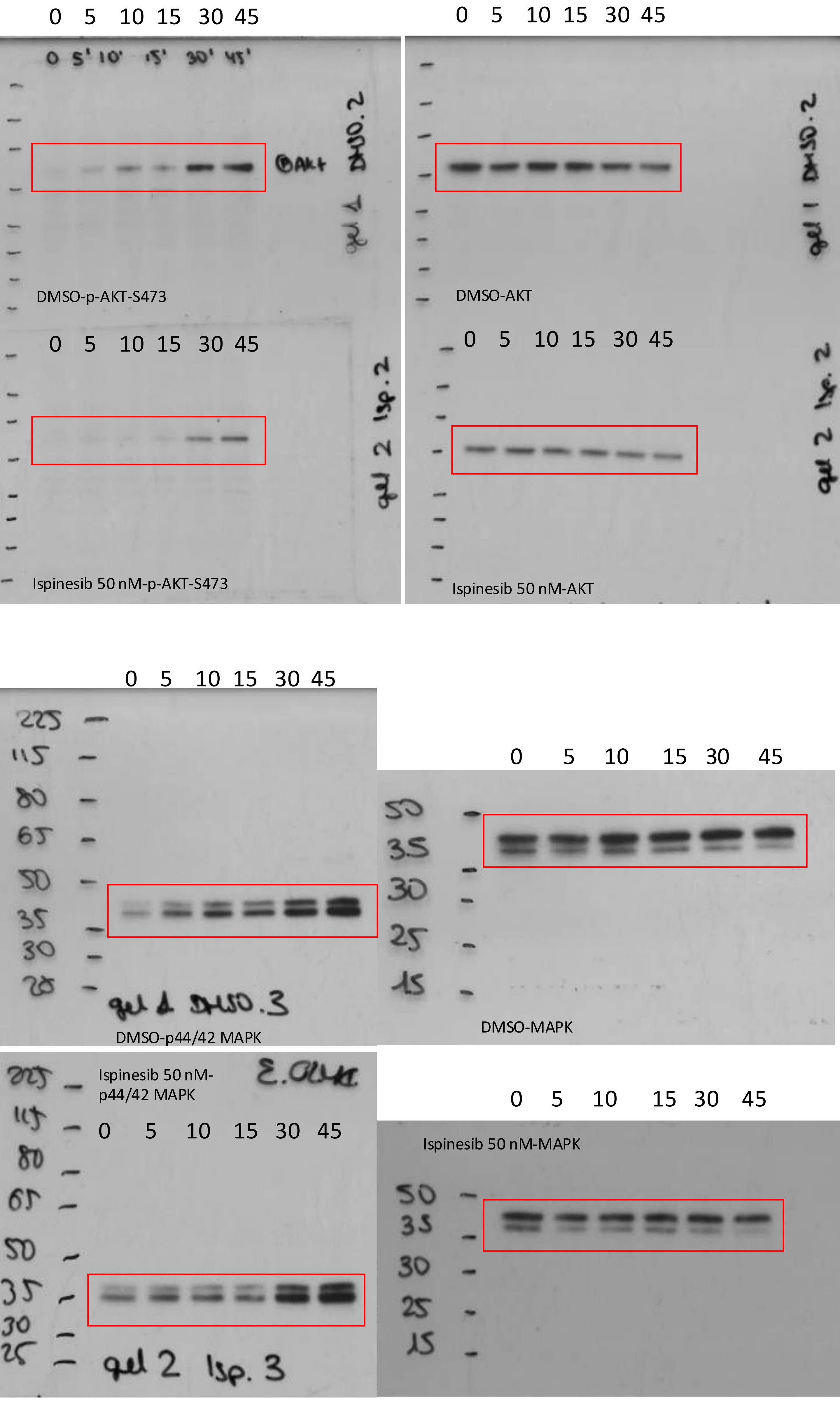

Full unedited gel for Figure 8E

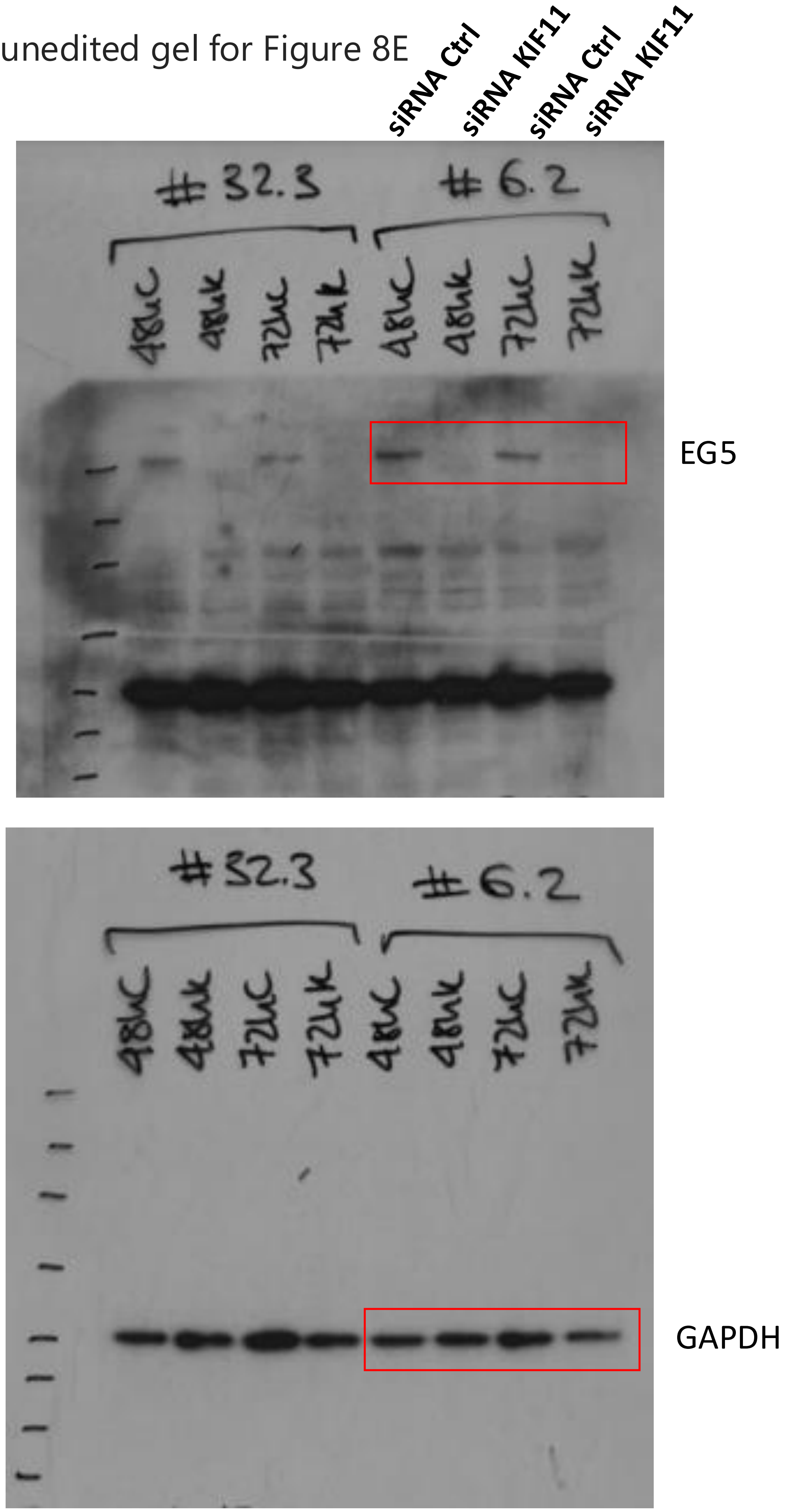

Full unedited gel for Figure 8E

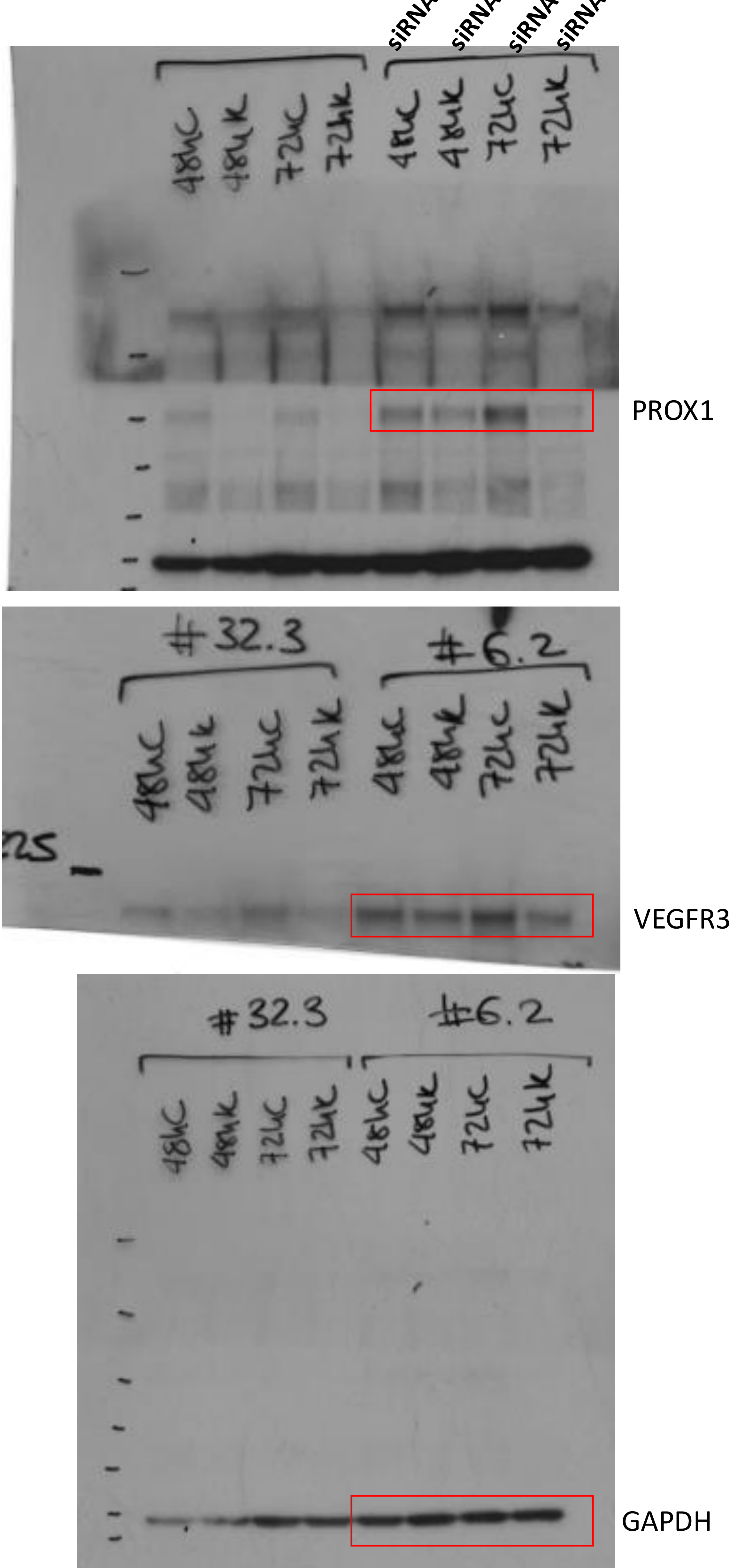

Full unedited gel for Figure S4A

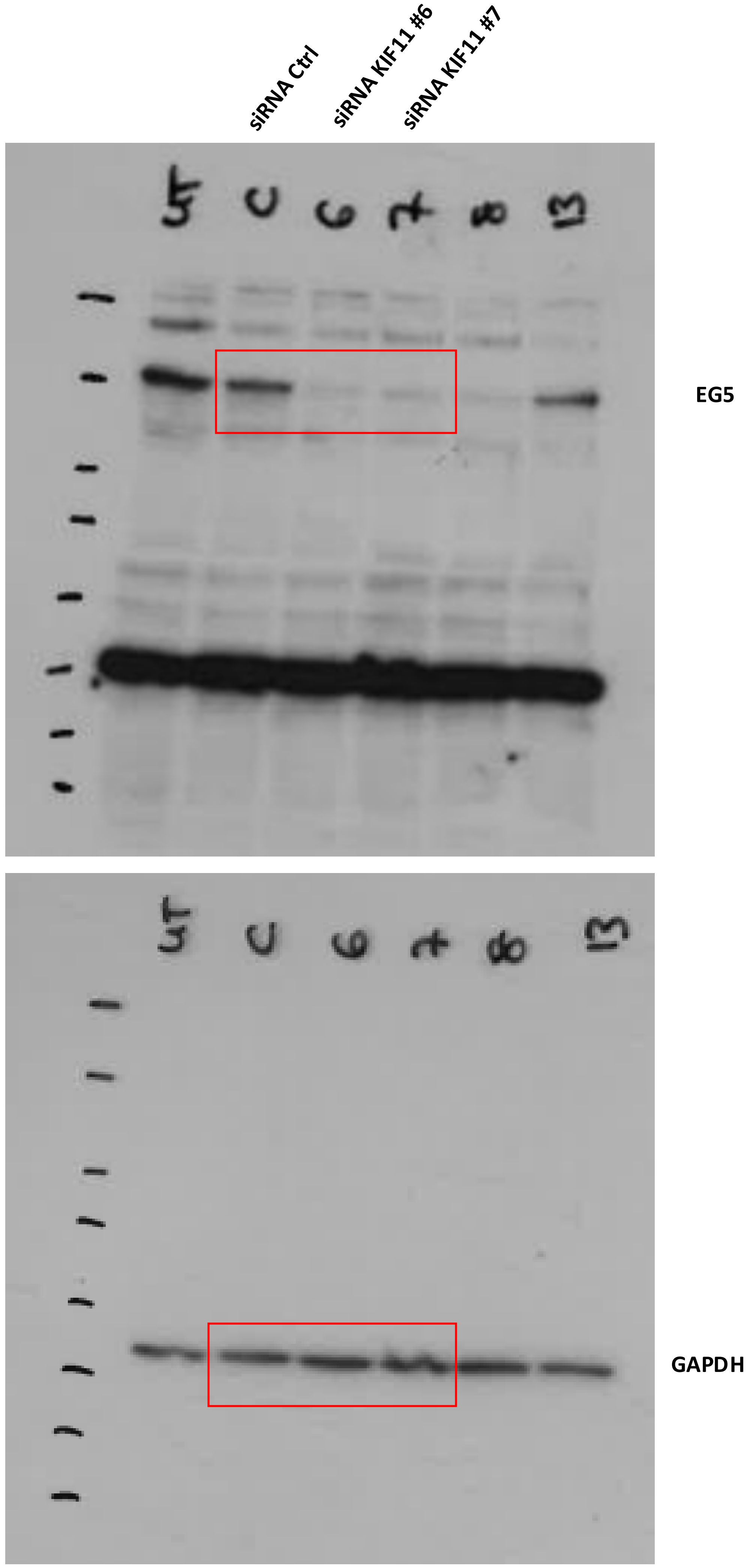

Supplement: Unedited blot and gel images [file jciinsight-11-177656-s217.pdf]
